# Inhibition of BCAT1-mediated cytosolic leucine metabolism regulates Th17 responses *via* the mTORC1-HIF1α pathway

**DOI:** 10.1038/s12276-024-01286-z

**Published:** 2024-08-01

**Authors:** Yeon Jun Kang, Woorim Song, Su Jeong Lee, Seung Ah Choi, Sihyun Chae, Bo Ruem Yoon, Hee Young Kim, Jung Ho Lee, Chulwoo Kim, Joo-Youn Cho, Hyun Je Kim, Won-Woo Lee

**Affiliations:** 1 Laboratory of Autoimmunity and Inflammation (LAI), Department of Biomedical Sciences, Seoul National University College of Medicine, Seoul, 03080 Republic of Korea; 2 Department of Microbiology and Immunology, Seoul National University College of Medicine, Seoul, 03080 Republic of Korea; 3 Department of Biomedical Sciences, Seoul National University Graduate School, Seoul, 03080 Korea; 4 Department of Clinical Pharmacology and Therapeutics, Seoul National University, College of Medicine and Hospital, Seoul, 03080 Republic of Korea; 5 Department of Microbiology, Institute for Viral Diseases, Korea University College of Medicine, Seoul, 02841 Republic of Korea; 6 Seoul National University Cancer Research Institute, Institue of Endemic Diseases and Ischemic/Hypoxic Disease Institute, Seoul National University Medical Research Center, Seoul National University Hospital Biomedical Research Institute, Seoul, 03080 Republic of Korea

**Keywords:** Interleukins, Chronic inflammation

## Abstract

Branched-chain amino acids (BCAAs), particularly leucine, are indispensable AAs for immune regulation through metabolic rewiring. However, the molecular mechanism underlying this phenomenon remains unclear. Our investigation revealed that T-cell receptor (TCR)-activated human CD4^+^ T cells increase the expression of BCAT1, a cytosolic enzyme responsible for BCAA catabolism, and SLC7A5, a major BCAA transporter. This upregulation facilitates increased leucine influx and catabolism, which are particularly crucial for Th17 responses. Activated CD4^+^ T cells induce an alternative pathway of cytosolic leucine catabolism, generating a pivotal metabolite, β-hydroxy β-methylbutyric acid (HMB), by acting on BCAT1 and 4-hydroxyphenylpyruvate dioxygenase (HPD)/HPD-like protein (HPDL). Inhibition of BCAT1-mediated cytosolic leucine metabolism, either with BCAT1 inhibitor 2 (Bi2) or through BCAT1, HPD, or HPDL silencing using shRNA, attenuates IL-17 production, whereas HMB supplementation abrogates this effect. Mechanistically, HMB contributes to the regulation of the mTORC1-HIF1α pathway, a major signaling pathway for IL-17 production, by increasing the mRNA expression of HIF1α. This finding was corroborated by the observation that treatment with L-β-homoleucine (LβhL), a leucine analog and competitive inhibitor of BCAT1, decreased IL-17 production by TCR-activated CD4^+^ T cells. In an in vivo experimental autoimmune encephalomyelitis (EAE) model, blockade of BCAT1-mediated leucine catabolism, either through a BCAT1 inhibitor or LβhL treatment, mitigated EAE severity by decreasing HIF1α expression and IL-17 production in spinal cord mononuclear cells. Our findings elucidate the role of BCAT1-mediated cytoplasmic leucine catabolism in modulating IL-17 production *via* HMB-mediated regulation of mTORC1-HIF1α, providing insights into its relevance to inflammatory conditions.

## Immune regulation: leucine’s role in Th17 response modulation

T-cell, a type of infection-fighting white blood cell, alter their metabolic process, relying heavily on amino acids, the building blocks of proteins. This study investigates how T cells use the amino acid leucine to power their response. Researchers conducted experiments with human T-cell and a mouse model of autoimmune disease, a condition where the body attacks its own cells. They studied how leucine’s metabolic process affects T-cell function. The study discovered that a specific process involving leucine’s metabolic pathway in T cells is vital for their ability to produce IL-17. Blocking a crucial enzyme reduced IL-17 production and eased symptoms in a mouse model of autoimmune disease. These findings underline the importance of leucine’s metabolic process in T-cell function and suggest a potential target for treating autoimmune diseases more effectively, offering hope for new treatments.

This summary was initially drafted using artificial intelligence, then revised and fact-checked by the author.

## Introduction

T-cell activation is central to adaptive immune responses for host defense. This process is coupled with bioenergetic and biosynthetic demands to support the proliferation, differentiation, and cytokine production of T cells in response to antigens^1,2^. Activated T cells undergo dynamic changes in their metabolism through several mechanisms, including an increase in glucose uptake and glucose metabolism, mitochondrial function, amino acid uptake, and lipid synthesis^2,3^. Metabolic status decisively governs T-cell functional plasticity^4,5^. Accumulating evidence has demonstrated that amino acids are key nutrients for maintaining the high metabolic status of activated immune cells and supporting a variety of immune cell functions^6,7^. The expression of amino acid transporters such as SLC7A5 (also called L-type amino acid transporter 1 [LAT1]) is upregulated to fulfill the specific amino acid requirements of activated immune cells^7,8^. In addition to their fundamental role as building blocks for protein synthesis, amino acids also participate in other intracellular metabolic pathways by directly acting as nutrient signals to regulate signaling pathways or by converting into metabolic intermediates^6^.

Branched-chain amino acids (BCAAs), including leucine, isoleucine and valine, are essential for normal growth and development due to their ability to promote protein synthesis, mainly through the activation of the mTOR signaling pathway^9^. In immune cells, mTOR plays a critical role as a regulator of cellular metabolism, influencing multiple functions, including proliferation, differentiation, and effector responses^10,11^. Branched-chain aminotransferases (BCATs), the initial enzymes of BCAA catabolism, reversibly transaminate BCAAs to branched-chain α-keto acids (BCKAs), which undergo oxidative decarboxylation by the branched-chain α-keto acid dehydrogenase complex (BCKDC) to form CoA derivatives, which act as substrates of the TCA cycle to generate ATP^12,13^. Two isozymes of BCATs exist: mitochondrial BCAT (BCATm or BCAT2) and cytosolic BCAT (BCATc or BCAT1). BCAT2 is expressed in most tissues except the liver and is thus regarded as a major enzyme for BCAA catabolism, whereas the expression of BCAT1 is limited to the nervous system^14^. Due to its ubiquitous expression and primary role in BCAA metabolism, a large number of studies have been conducted on the role of BCAT2 in this process. Interestingly, recent studies have shown that BCAT1 is highly expressed in activated T cells and macrophages and is involved in regulating inflammatory responses^15,16^, suggesting its possible role in modulating metabolic programming in activated immune cells. However, how BCAT1-mediated metabolism of BCAAs, especially leucine, influences T-cell-mediated immune responses and the underlying molecular mechanism remain unclear.

Our data revealed that the BCAT1-mediated leucine metabolite HMB predominantly augments Th17 responses *via* regulation of the mTORC1-HIF1α pathway, a major signaling pathway for IL-17 production. Moreover, BCAT1 inhibition with BCATc inhibitor 2 (Bi2) alleviates disease severity in an EAE mouse model by decreasing HIF1α expression and IL-17 production in T cells in the spinal cord. Our findings suggest that SLC7A5-mediated influx and BCAT1-mediated leucine catabolism play crucial roles in modulating CD4^+^ T-cell responses, especially IL-17 production, *via* the regulation of HIF1α. Furthermore, this mechanism might be related to various inflammatory conditions.

## Materials and methods

### Preparation and culture of human T cells

The study protocols were reviewed and approved by the IRB of Seoul National University Hospital. Peripheral blood was drawn from healthy controls (HCs) after written informed consent was obtained. The methods were performed following the approved guidelines. Peripheral blood mononuclear cells (PBMCs) were isolated from peripheral blood by density gradient centrifugation (Biocoll separating solution; BIOCHROM Inc., Cambridge, UK). Total, naive, and memory CD4^+^ T cells were negatively separated from CD14^+^ monocyte-depleted PBMCs using MojoSort^TM^ Human Total CD4^+^ T cells, CD4^+^ naive T cells, and CD4^+^ memory T-cell isolation kits, respectively (BioLegend, San Diego, CA). Purified T cells were cultured in RPMI 1640 medium supplemented with 10% fetal bovine serum (FBS), 1% penicillin/streptomycin, and 1% L-glutamine (henceforth, complete RPMI 1640). Cells were stimulated with anti-CD3/CD28-coated microbeads 1:10 (Dynabeads T-Activator CD3/CD28, Thermo Fisher Scientific, Waltham, MA) in the presence of the indicated chemical inhibitors or reagents, including 2-amino-2-norbornanecarboxylic acid (BCH), JPH203, acetate, MG132, VH298, cobalt chloride (CoCl_2_), cycloheximide (CHX) (all from Sigma-Aldrich, St. Louis, MO), BCAT1 inhibitor 2 (Bi2; Cayman Chemical, Ann Arbor, MI), 3-hydroxy-3-methylbutyric acid (HMB; Thermo Fisher Scientific, Waltham, MA), and L-β-homoleucine (LβhL, Santa Cruz Biotechnology, Heidelberg, Germany). In some experiments, custom-made media depleted of five essential amino acids (leucine, valine, isoleucine, phenylalanine, and methionine) were used (Welgene, Kyungsan, Republic of Korea). All amino acids (L-leucine, L-valine, L-isoleucine, L-phenylalanine, and L-methionine) used for the replenishing experiments were purchased from Sigma‒Aldrich.

### Quantitative RT‒PCR

Total RNA was prepared using TRIzol reagent (Life Technologies, Grand Island, NY), followed by cDNA synthesis (Promega, Madison, WI), and then real-time quantitative RT‒PCR was performed with a CFX system (Bio-Rad, Hercules, CA) using SensiFAST SYBR® Lo-ROX (Bioline, London, UK). The sequences of the primers used in this study are shown in Supplementary Table 1. Gene expression levels were normalized to the expression of ACTINB using the comparative CT method (*ΔΔ*CT).

### Enzyme-linked immunosorbent assay (ELISA)

The amounts of IL-17A and IFN-γ in the culture supernatant were quantified using commercially available human ELISA kits according to the manufacturer’s instructions (IL-17A ELISA kits from eBioscience and Human IFN-γ ELISA MAX Deluxe from BioLegend). Optical density was measured using an Infinite M200 instrument (Tecan, Männedorf, Switzerland).

### Immunoblot analysis

Cells were incubated in radioimmunoprecipitation assay (RIPA) buffer (150 mM NaCl, 10 mM Na_2_HPO_4_, pH 7.2, 1% Nonidet P-40, and 0.5% deoxycholate) containing phenylmethylsulfonyl fluoride (PMSF; Millipore Sigma, Burlington, MA), EDTA, and a protease and phosphatase inhibitor cocktail (Thermo Fisher Scientific) to extract the total protein. The proteins were separated on an 8–10% SDS-polyacrylamide gel and blotted onto a polyvinylidene difluoride (PVDF) membrane (Bio-Rad, Hercules, CA), followed by blocking for 1 h with 5% BSA in Tris-buffered saline solution (TBS) containing 0.1% Tween 20. The membrane was incubated overnight at 4 °C with anti-human SLC7A5, anti-BCAT, anti-phospho-p70S6K, anti-p70S6K, and anti-HIF-1α (all from Cell Signaling Technology, Danvers, MA) primary Abs followed by incubation with the HRP-conjugated secondary Ab for 1 h at RT. The membranes were developed using SuperSignal West Femto Maximum Sensitivity Substrate or SuperSignal West Pico PLUS Chemiluminescent Substrate system (Thermo Fisher Scientific).

### Flow cytometry analysis

To analyze the proliferation and cytokine production of T cells, carboxyfluorescein succinimidyl ester (CFSE; Invitrogen, Waltham, MA, USA) dilution assay and intracellular cytokine staining (ICS) were conducted using the antibodies listed in Supplementary Table 2 and a BD LSRFortessa cell analyzer as described previously^17^.

### ^3^H-amino acid uptake assay

The cells were incubated in HBSS for 10 min after the culture media was removed. The ^3^H-leucine or ^3^H-methionine (Perkin Elmer, Waltham, MA) uptake assay was initiated by incubating the cells in HBSS containing 0.5–1 μCi for 15 min. The cells were detached with 1 M NaOH after three washes with ice-cold HBSS. The radioactivity was measured using the beta scintillation counter MicroBeta® (Perkin Elmer).

### Lentivirus production and transduction of human CD4^+^ memory T cells

To silence BCATc, HPD, and HPDL expression in human CD4^+^ memory T cells, we used BCATc, HPD, or HPDL human shRNA plasmids containing the GFP reporter gene (Origene, Rockville, MD; Cat# TL314498 for BCATc, TL312348 for HPD, TL304319 for HPDL, and TR30021 for control). Lentivirus was produced by transfection of the lentiviral vector, along with psPAX2 (#12260) and pMD2. G (#12259) (both from Addgene, Watertown, MA) expression vectors into 293FT cells using Fugene (Promega, Madison, WI). Lentiviral particles were collected 48 and 72 h after transfection, filtered through a 0.45 µm syringe filter (Millipore), concentrated using Peg-it solution (System Biosciences, Palo Alto, CA), and titrated on 293FT cells. For lentiviral transduction, purified CD4^+^ memory T cells were activated with anti-CD3/CD28-coated microbeads and transduced with a lentiviral vector expressing scrambled control or target shRNA at a multiplicity of infection of 10 in the presence of 8 mg/ml polybrene (Sigma). After 24 h, shRNA^+^ cells expressing GFP were sorted with a BD FACSAria™ III Cell Sorter (BD Bioscience) and activated with anti-CD3/CD28-coated microbeads for another 3 days.

### Cholesterol assay

The total amount of intracellular cholesterol in cultured cells was determined using the fluorometric Amplex™ Red Cholesterol Assay Kit (Thermo Fisher Scientific) according to the manufacturer’s instructions.

### ScRNA-seq analysis

Freshly purified CD4^+^ memory T cells were stimulated with anti-CD3/CD28-coated microbeads in the absence or presence of Bi2 for 72 h. Samples from four different donors were multiplexed with a hashtag oligo for 30 min at 4 °C using Totalseq^TM^ anti-human hashtag antibody (Biolegend, USA) under three experimental conditions: TCR-stimulated cells with Bi2, TCR-stimulated cells without Bi2, and no TCR stimulation. Tagged cells were pooled and loaded into the Chromium system (10x Genomics, Pleasanton, CA) for encapsulation into a single droplet targeting ~40,000 cells for each GEM (Gel Beads-in-emulsion). A Chromium Single Cell 5’ Kit (10x Genomics) was used to generate the scRNA-seq and TCR libraries according to the manufacturer’s instructions. Demultiplexing and read alignment to the combined human genome were performed using Cell Ranger (v6.1.2).

### EAE induction

The female C57BL/6 mice (12 weeks old) used for the induction of EAE were purchased from KOATECH (Pyeongtaek, Gyeonggi, Republic of Korea). All mice were housed and maintained in a pathogen-free facility at Seoul National University (SNU) College of Medicine. All experiments were approved by the SNU IACUC. Mice were injected intraperitoneally with 10 mg/kg BCAT1 inhibitor 2 (Bi2) in 200 μl of sterile PBS 3 times per week until 14 days postimmunization. To induce EAE, mice received bilateral subcutaneous injections of a total of 200 μg of MOG 35–55 peptide (GeneScript, Piscataway, NJ) emulsified in CFA (BD Biosciences, San Jose, CA), followed by i.p. injection of 250 ng pertussis toxin (PTX) (List Biological Labs, Campbell, CA) 4 and 24 h later. The clinical scores of EAE mice were assessed daily.

### LC‒MS analysis of intracellular alpha-ketoisocarproic acid

A total of 1 × 10^6^ HepG2 cells were quenched with 200 µl of ice-cold 50% methanol in distilled water. After centrifugation, the supernatant was transferred to a new tube, and 200 μl of ice-cold 50% methanol was added to the remaining pellet. After vortexing and centrifugation, the supernatant was collected and combined with the previously collected supernatant. The samples were stored at −80 °C until analysis. Before analysis, the samples were thawed and filtered through 0.2 µm syringe filters and then transferred to LC‒MS tubes.

Liquid chromatography–orbitrap mass spectrometry using a Thermo Fisher Scientific Vanquish^TM^ UPLC system coupled with a Thermo Fisher Scientific Orbitrap Exploris^TM^ 120 was used to analyze the levels of alpha-ketoisocarproic acid (α-KIC). An ACQUITY UPLC HSS T3 column (100 Å, 1.8 µm, 2.1 mm × 100 mm) was used for separation. HPLC grade water was used as mobile phase A, and HPLC grade methanol was used as mobile phase B. The column was maintained at 40 °C, the injection volume was 5 µl, and the gradient was carried out at a flow rate of 0.3 ml min^−1^. The samples were eluted with a linear gradient (curve = 5) consisting of 5–30% B for 0–3 min, 90% B for 3.5 min, a constant gradient of 90% B for 3 min, 5% B for 7 min, and 5% B for 3 min for re-equilibration, as recommended by previous research^18^.

Mass analysis was performed with the following parameters: spray voltage, 4000 V for positive mode and 3000 V for negative mode; sheath gas flow, 40 L h^−1^; aux gas flow, 10 L h^−1^; sweep gas flow, 1 L h^−1^; ion transfer tube temperature, 340 °C; and vaporizer temperature, 350 °C. α-KIC was specifically determined with a targeted single ion monitoring (tSIM) scan, followed by a data-dependent MS2 (ddMS^2^) scan with a targeted mass filter. For both metabolites, the isolation window was set at 2 (*m/z*), the Orbitrap resolution was set at 120,000 for tSIM and 15,000 for ddMS^2^, the RF lens was set at 70%, and the scan range was set at 150–2000 (*m/z*) for mass scan and 40–150 (*m/z*) for ddMS^2^. The collision energy was set to 20 eV for α-KIC. The qualifier ion was chosen as follows for identification: α-KIC (129.0556 > 69.0346). For peak detection and area calculation, the Thermo Fisher Scientific FreeStyle program was used.

### Statistical analysis

Two-tailed unpaired *t* tests, Mann‒Whitney U tests, one-way ANOVAs with Kruskal–Wallis tests, one-way ANOVAs with Tukey’s tests, or two-way ANOVAs were used to analyze the data using Prism 9 software (GraphPad Software Inc., La Jolla, CA), as indicated in the figure legends. *p* values less than 0.05 were considered to indicate statistical significance.

## Results

### The expression of BCAT1 and SLC7A5 is upregulated by TCR-stimulated CD4^+^ T cells

**Fig. 1:**
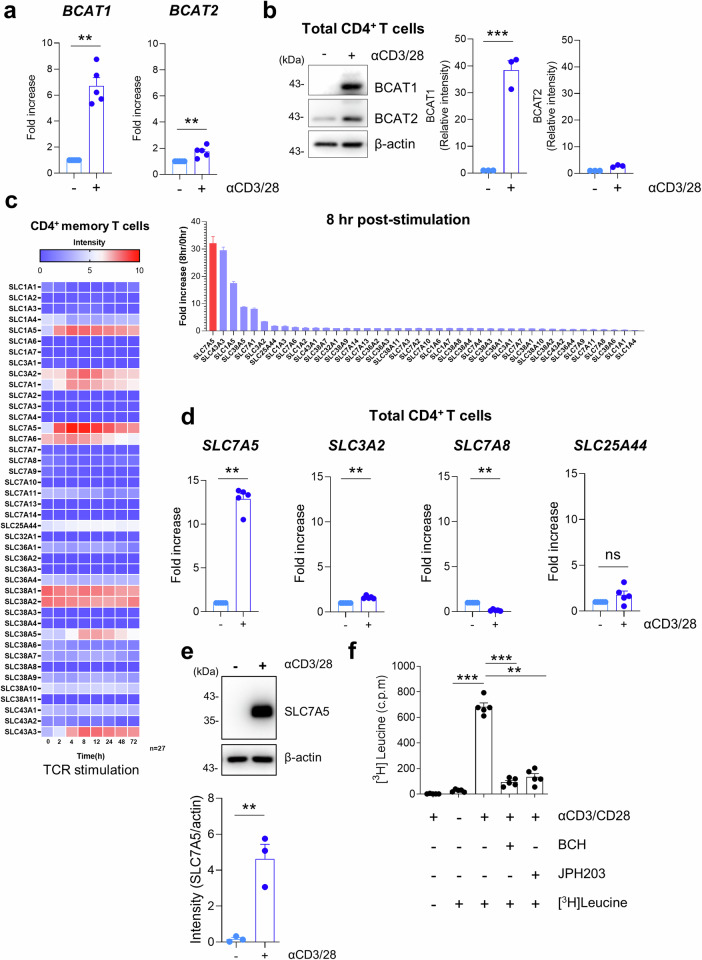
TCR stimulation induces the expression of BCAT1 and SLC7A5 in human CD4^+^ T cells. **a**, **b** The mRNA and protein expression of BCAT1 and BCAT2 in human CD4^+^ T cells from healthy controls (HCs) was analyzed by RT‒qPCR (A; *n* = 5) and immunoblotting (B; *n* = 3) at 24 h with or without TCR stimulation. **c** Public RNA-seq data (GEO No: GSE140244) were analyzed to examine the expression of 42 amino acid transporters at the indicated time points in TCR-stimulated human CD4^+^ memory T cells. Heatmap analysis (left) and fold change in the expression (right) of amino acid transporters at the indicated times after TCR stimulation. **d** The expression of major BCAA transporters was validated by RT‒qPCR at 24 h poststimulation in human CD4^+^ T cells from HCs (*n* = 5). **e** The protein expression of SLC7A5 in human CD4^+^ T cells from HCs was analyzed at 24 h with or without TCR stimulation (*n* = 3). **f** Uptake of ^3^H-leucine by TCR-stimulated human CD4^+^ T cells in the presence of 50 mM BCH and 10 μM JPH203 (*n* = 5). The graphs show the means ± SEMs. *p < 0.05, **p < 0.01, and ***p < 0.001 according to the Mann‒Whitney *U* test (**a**, **d**, **f**), two-tailed unpaired *t* test (**b**, **e**), or one-way ANOVA wi*t*h Tukey’s test (**f**).

To explore the immunoregulatory role of BCATs in T-cell responses, we first examined BCAT expression in TCR-activated T cells derived from healthy controls (HCs). Cytosolic BCAT1 mRNA and protein expression was markedly increased in CD4^+^ T cells upon TCR stimulation, and mitochondrial BCAT2 was also slightly upregulated in these cells (Fig. 1a, b). Since BCAT1 facilitates the reversible transamination of BCAAs, making it the first enzyme in the degradation pathway^12,16^, we next questioned whether TCR stimulation induces the expression of BCAA-specific transporters. Analysis of public RNA-Seq data (GSE140244) and confirmatory qPCR revealed that in human CD4^+^ T cells, the expression of several amino acid transporters, including BCAA-specific SLC7A5 and SLC3A2, was dependent on TCR stimulation (Fig. 1c, d)^19^. As shown in Fig. 1e, TCR stimulation predominantly upregulated SLC7A5 expression in human primary CD4^+^ T cells but not in CD8^+^ T cells under the same culture conditions (Supplementary Fig. 1a). Moreover, TCR-activated CD4^+^ T cells efficiently incorporated ^3^H-labeled leucine, a BCAA, and this uptake was greatly reduced in the presence of JPH203, a specific inhibitor of SLC7A5 (LAT1), or BCH (2-amino-2-norbornanecarboxylic acid), a general inhibitor of LATs (Fig. 1f). In addition, TCR-activated CD4^+^ T cells also mediated the uptake of methionine, which is primarily transported by SLC7A5, suggesting that TCR-induced SLC7A5 functions well (Supplementary Fig. 1b). Our findings showed that TCR stimulation upregulates the expression of SLC7A5 and BCAT1 to increase the uptake and metabolism of BCAAs by human CD4^+^ T cells.

### SLC7A5-mediated leucine influx influences CD4^+^ T-cell responses

**Fig. 2:**
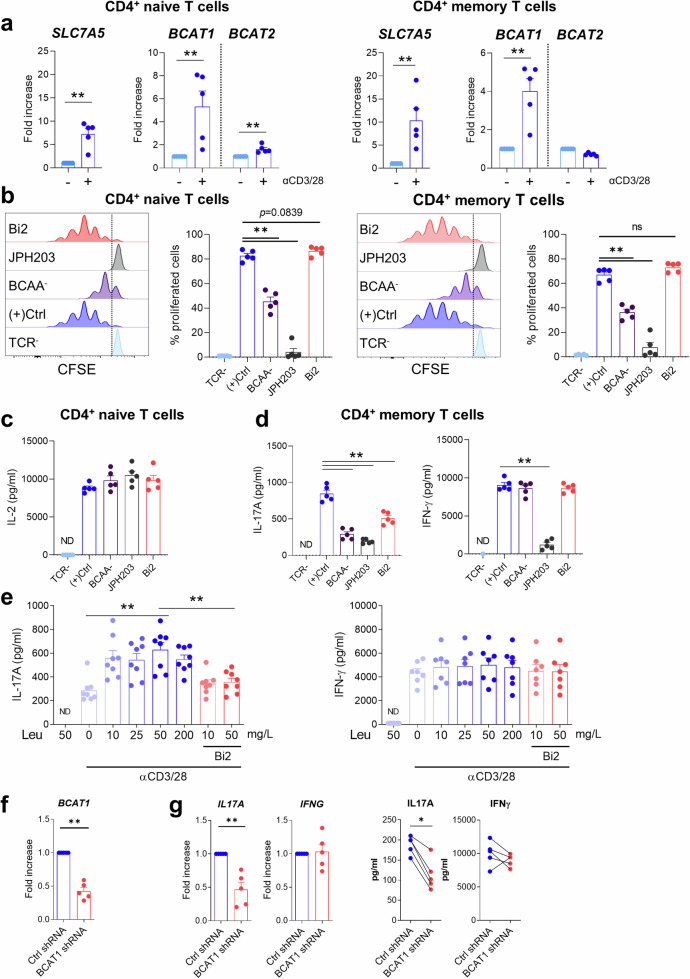
SLC7A5-mediated leucine influx regulates Th17 responses. **a** mRNA expression of SLC7A5, BCAT1, and BCAT2 was analyzed by RT‒qPCR at 24 h poststimulation in human naive and memory CD4^**+**^ T cells (*n* = 5). **b** CFSE-labeled naive and memory CD4^+^ T cells were stimulated with anti-CD3/CD28-coated microbeads for 4 days with or without BCAAs, Bi2 (10 μM), or JPH203 (10 μM) (*n* = 5). The proportion of proliferating cells was measured by CFSE dilution assay. **c**, **d** The amount of cytokines in the culture supernatant of TCR-stimulated CD4^+^ naive T cells (**c**) and CD4^+^ memory T cells (**d**) stimulated with anti-CD3/CD28-coated microbeads for 3 days under the indicated conditions (*n* = 5). **e** The amount of IL-17A (left) and IFN-γ (right) in culture supernatant from TCR-stimulated CD4^+^ memory T cells supplemented with leucine and Bi2 (*n* = 7–8). **f**, **g** Freshly isolated human CD4^+^ memory T cells were activated and infected with GFP lentivirus containing BCAT1 shRNA for 24 h. shRNA^+^ cells expressing GFP were sorted and cultured for another 3 days with TCR stimulation. BCAT1 expression in sorted shRNA^+^ cells (*n* = 5) (**f**). The mRNA (left) and protein (right) levels of IL-17 and IFN-γ in the culture supernatant were analyzed by RT‒qPCR (*n* = 5) and ELISA (*n* = 5) (**g**). The graphs show the means ± SEMs. *p < 0.05 and **p < 0.01 according to the Mann‒Whitney *U* test.

We next investigated the effects of SLC7A5-mediated BCAA influx on CD4^+^ T-cell responses. SLC7A5 and BCAT1 expression was elevated to a similar degree in TCR-activated naive and memory CD4^+^ T cells (Fig. 2a). The proliferation of these cells was also comparable under various conditions that cause changes in SLC7A5-mediated influx and metabolism of BCAAs. While JPH203 completely blocked the proliferation of CD4^+^ naive and memory T cells, the depletion of BCAAs in culture media resulted in a partial reduction in proliferation (Fig. 2b). In contrast, no effect on proliferation was observed in TCR-stimulated CD4^+^ naive and memory T cells pretreated with BCATc inhibitor 2 (Bi2), a BCAT1-specific inhibitor (Fig. 2b)^20^. The inhibitory potential of Bi2 was evaluated by quantifying the expression of alpha-ketoisocaproate (α-KIC), a metabolite of BCAT1, in HepG2 cells, a human liver cell line known for its consistent and abundant BCAT1 expression (Supplementary Fig. 2)^21^. The effects of changes in SLC7A5-mediated influx and metabolism of BCAAs on cytokine production differed depending on the CD4^+^ T-cell subset and cytokine profile. The production of IL-2 by CD4^+^ naive T cells was not influenced by JPH203 or Bi2 treatment or by BCAA depletion, whereas IL-17A and IFN-γ production by CD4^+^ memory T cells was greatly reduced following JHP203 treatment (Fig. 2c, d). Notably, BCAA depletion and Bi2 treatment preferentially repressed IL-17A production (Fig. 2d), suggesting that intracellular BCAAs and their metabolites are pivotal for IL-17A production in CD4^+^ memory T cells. To further determine which BCAA is important for regulating proliferation and cytokine production, TCR-stimulated CD4^+^ memory T cells were cultured in leucine-, isoleucine-, or valine-depleted media. While depletion of each BCAA had a minimal effect on cell survival, an inhibitory effect on the proliferation and cytokine production of CD4^+^ memory T cells was observed, with the exception of IFN-γ production in leucine-depleted media (Supplementary Fig. 3a–c). Thus, leucine depletion tends to influence the production of IL-17A. Given that leucine is involved in regulating the activity of mTORC1, a major signaling pathway for IL-17A expression^22,23^, we decided to focus on the role of SLC7A5-mediated leucine influx and its metabolites by BCATs in Th17 responses. Leucine depletion in culture media significantly reduced the production of IL-17A, but not IFN-γ, by CD4^+^ memory T cells upon TCR stimulation compared to that in conventional complete RPMI-1640 media supplemented with 50 mg/ml leucine (Fig. 2e). Blockade or silencing of BCAT1 inhibited the production of IL-17A even in medium containing sufficient leucine (Fig. 2e–g). The phosphorylation of S6 kinase (S6K), which is an important downstream substrate of mTORC1 for IL-17 production, was dependent on the presence of leucine in the media (Supplementary Fig. 3d). These data demonstrate that SLC7A5-mediated influx and cytosolic metabolism of BCAAs are important for the regulation of IL-17A production in human CD4^+^ memory T cells.

### BCAT1-mediated leucine metabolite HMB regulates Th17 responses

**Fig. 3:**
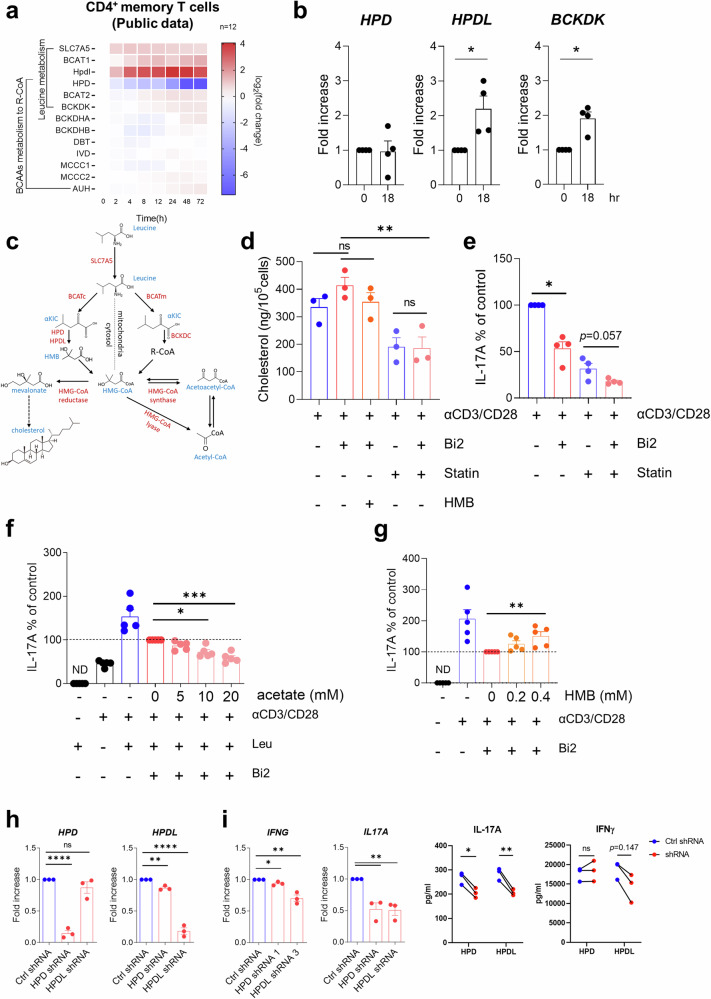
HMB, a BCAT1-mediated leucine metabolite, is involved in the regulation of Th17 responses. **a** Public RNA-seq data (GSE140244) were analyzed to examine the expression of BCAA catabolic enzymes at the indicated times in TCR-stimulated human CD4^+^ memory T cells. Heatmap analysis illustrating the time course of changes in the expression of major BCAA catabolic enzymes. **b** The expression of the HPD, HPDL, and BCKDK in human CD4^+^ T cells was validated by RT‒qPCR at 24 h poststimulation (*n* = 4). **c** Scheme of cytosolic leucine metabolism. **d** CD4^+^ memory T cells were pretreated with Bi2 (10 μM), a statin (10 μM), or HMB (0.4 mM) for 1 h and stimulated with anti-CD3/CD28-coated microbeads for 3 days. The accumulation of cholesterol in cell lysates was measured (*n* = 3). **e** The amount of IL-17A in the culture supernatant from TCR-stimulated CD4^+^ memory T cells was measured on Day 3 by ELISA (*n* = 4). **f** TCR-stimulated CD4^+^ memory T cells were cultured for 3 days with Bi2 and the indicated concentration of acetate. The amount of IL-17A was measured by ELISA (*n* = 5). **g** The amount of IL-17A in the culture supernatant of CD4^+^ memory T cells supplemented with HMB was measured by ELISA (*n* = 5). **h**, **i** Freshly isolated human CD4^+^ memory T cells were activated and infected with GFP lentivirus containing HPD or HPDL shRNA for 24 h. shRNA^+^ cells expressing GFP were sorted and cultured for another 3 days with TCR stimulation. Expression of the indicated genes in sorted shRNA^+^ cells (*n* = 3) (**h**). The mRNA (left) and protein (right) levels of IL-17 and IFN-γ in the culture supernatant were analyzed by RT‒qPCR (*n* = 3) and ELISA (*n* = 3) (**i**). The graphs show the means ± SEMs. *p < 0.05, **p < 0.01, ***p < 0.001, and ****p < 0.0001 according to the Mann‒Whitney *U* test (**b**, **e**), one-way ANOVA with the Kruskal‒Wallis test (**d**, **f**, **g**), or two-tailed unpaired *t* test (**h**, **i**), respectively.

Reanalysis of public RNA-Seq data revealed that the expression of BCAA metabolism-related genes, particularly leucine, was induced in TCR-activated CD4^+^ memory T cells in humans (Fig. 3a). qPCR confirmed that TCR stimulation increased the expression of the HPDL and BCKDK genes. The 4-hydroxyphenylpyruvate dioxygenase-like protein encoded by the HPDL gene has dioxygenase activity similar to that of HPD^24^ and is involved in the generation of β-hydroxy-β-methyl butyrate (HMB), a leucine metabolite^25^ (Fig. 3a, b). In cytosolic leucine metabolism pathways, HMB is known to be further metabolized into HMG-CoA and to participate in the de novo synthesis of cholesterol and acetyl-CoA (Fig. 3c), which regulate IL-17A production in CD4^+^ T cells^26–28^. Thus, we examined the effect of these two leucine end products on IL-17 production in CD4^+^ memory T cells. Cholesterol was slightly increased following blockade of BCAT1 with Bi2 treatment, and this increase was suppressed by HMB supplementation (Fig. 3d). Treatment with a statin, a mevalonate synthesis inhibitor, led to significant reductions in cholesterol and IL-17A production, as previously reported^27^. Notably, Bi2 further decreased IL-17A production independent of cholesterol levels in statin-treated CD4^+^ memory T cells (Fig. 3d, e). It has been demonstrated that acetyl-CoA enhances Th17 responses *via* epigenetic reprogramming^28^. We found that exogenous acetate dose-dependently decreased IL-17A production in Bi2-treated CD4^+^ memory T cells, which was expected to increase intracellular acetyl-CoA levels (Fig. 3f). In contrast, treatment with exogenous acetate upregulated the production of IFN-γ in TCR-stimulated CD4^+^ memory T cells treated with Bi2 (Supplementary Fig. 4a). HMB supplementation partially reversed the Bi2-mediated reduction in IL-17A production in CD4^+^ memory T cells (Fig. 3g) in a dose-dependent manner. However, IFN-γ production was not affected by the blockade of BCAT1 or HMB supplementation (Supplementary Fig. 4b). The effect of HMB was substantiated by silencing HPD and HPDL with shRNA, which significantly inhibited IL-17A production (Fig. 3h, i). These data indicate that HMB, a cytosolic leucine metabolite, contributes to augmented IL-17A production in human CD4^+^ T cells upon TCR stimulation.

### scRNA-seq analysis reveals unique signaling pathways involved in BCAT1-mediated regulation of IL-17A production

**Fig. 4:**
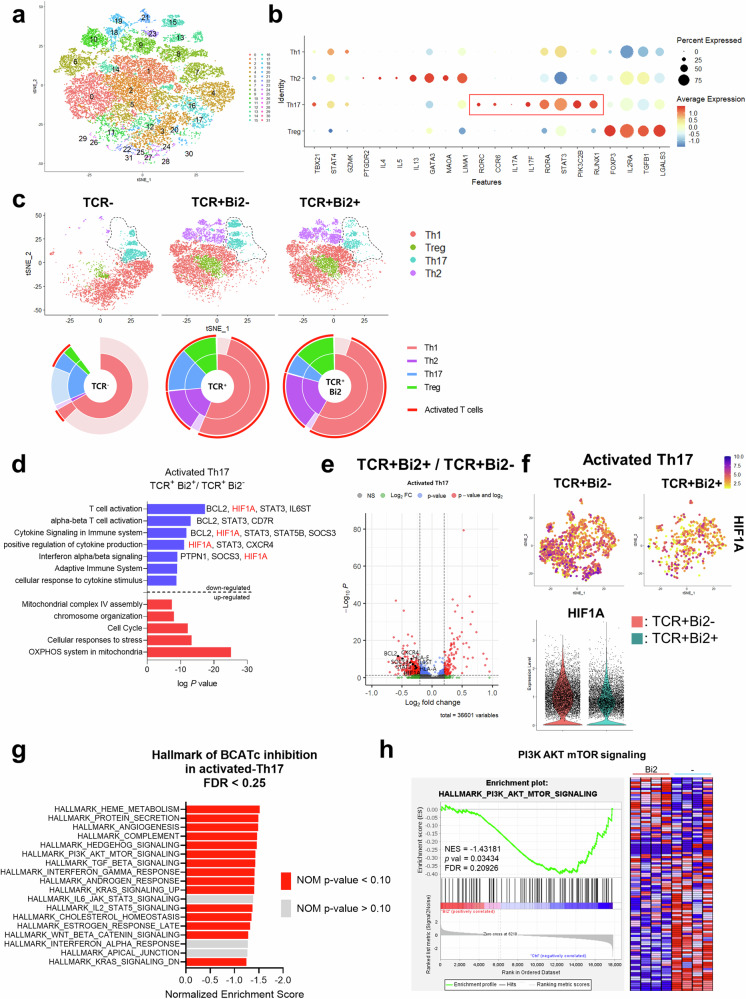
scRNA-seq analysis reveals unique signaling pathways involved in the BCAT1-mediated regulation of IL-17A production. Multiplex scRNA-seq analysis of human CD4^+^ memory T cells from three different groups was performed using Seurat in R software (version 4.3.0): no TCR stimulation (TCR-), TCR stimulation for 72 h without Bi2 (TCR+Bi2-), and TCR stimulation for 72 h with Bi2 (10 μM) (TCR+Bi2+), **a** Individual cells (28,651 cells) were color-coded based on the cluster (*n* = 31) in a *t*-distributed stochastic neighbor-joining (t-SNE) plot generated by unsupervised Seurat clustering. **b** Major CD4^+^ memory T-cell subsets were identified by canonical cell type marker expression. **c** *t*-SNE plots segregated on the basis of major CD4^+^ memory T-cell subsets (top, TCR-, *n* = 10,109 cells; TCR+Bi2-, *n* = 9506 cells; TCR+Bi2 +, *n* = 9036 cells). Dotted regions highlight Th17 cluster changes after TCR stimulation with Bi2 treatment. Pie charts showing relative Th subset abundances under different conditions (bottom). Activated T cells were annotated by the average expression of activation markers listed in Fig. S4A. **d** Pathway enrichment analysis of differentially expressed genes (DEGs) in activated Th17 cells between the TCR+Bi2- and TCR+Bi2+ groups. Representative genes in each pathway are shown. **e** Volcano plot of DEGs in activated Th17 cells between the TCR^+^Bi2^-^ and TCR^+^Bi2^+^ groups. Volcano plots were generated using the EnhancedVolcano package (version 1.16.0). **f** Expression of *HIF1A* in activated Th17 cells was projected onto the *t-*SNE plot (top) shown as feature plots (top). Violin plots showing the distribution of *HIF1A* expression levels, with dots representing individual cells (bottom). **g** Gene set enrichment analysis (GSEA) revealed 15 pathways enriched in 23,457 DEGs in the activated Th17 cluster with an FDR < 0.25. Red bars represent gene sets with a nominal *p* value < 0.1. **h** GSEA was used to examine the significantly enriched pathways. GSEA enrichment plot (left) and heatmap of downregulated DEGs (right) in the PI3K-AKT-mTOR signaling pathway in Bi2-treated cells. All transcripts within annotated genes were uploaded to a locally installed GSEA tool and compared with the hallmark gene sets.

To explore the molecular mechanism underlying the BCAT1-mediated regulation of IL-17A production, scRNA-seq analysis was performed in human CD4^+^ memory T cells with or without Bi2 at 72 h after TCR stimulation. Unsupervised clustering and *t*-distributed stochastic neighbor (*t*-SNE) plot analyses allowed us to determine cluster identities based on the expression of established markers (Fig. 4a and Supplementary Fig. 5a) and to successfully identify major CD4^+^ memory T-cell subsets, which were further grouped by the expression of human T-cell activation signatures (Fig. 4b, c). We observed that a subset classified as Th17 cells exhibited relatively heightened expression of leucine metabolism-related genes, including BCAT1, SLC7A5, and HPDL, in comparison to Th1 or Treg cells (Supplementary Fig. 5b) Our data show that the population of activated Th17 cells, which comprised 8, 13, and 15 clusters, was reduced following inhibition of cytosolic leucine metabolism with Bi2 treatment (Fig. 4c). To further analyze the biological significance of Bi2-mediated transcriptional changes in the Th17 subset, pathway enrichment analysis of differentially expressed genes (DEGs) was performed. We detected a decrease in T-cell activation, cytokine signaling, and cytokine production pathways following Bi2 treatment in the activated Th17 subset (Fig. 4d and Supplementary Table 3). These pathways were mainly driven by the downregulation of HIF1A, STAT3, and BCL2 (Fig. 4e, Supplementary Fig. 5c, d). The expression pattern of HIF1A was projected onto the *t*-SNE plot of activated Th17 cells (Clusters 8, 13, and 15). A feature plot and a violin plot illustrating that HIF1A expression is downregulated in activated Th17 cells following Bi2 treatment are shown (Fig. 4f). We next conducted gene set enrichment analysis (GSEA) on differentially expressed genes (DEGs) in Bi2-treated T cells to gain mechanistic insight into the genes identified *via* our scRNA-Seq analysis. There were 15 enriched pathways obtained from the 23,457 DEGs identified in the activated Th17 cluster of TCR-activated CD4^+^ memory T cells (*p* < 0.1 and FDR < 0.25) (Fig. 4g). Immune response-related GSEA pathways, such as complement, cholesterol homeostasis, PI3K-AKT-mTOR signaling, TGF-β signaling, IL-2/STAT5 signaling, and IFN-γ response, were included. Considering that leucine and its metabolites play a regulatory role in mTORC1 signaling activity in immune cells, we generated a GSEA enrichment plot and a heatmap of DEGs involved in the PI3K-AKT-mTOR signaling pathway that were downregulated in Bi2-treated cells (Fig. 4h). A negative normalized enrichment score (NES) indicated enrichment in activated Th17 cells. These data revealed that the mTORC1-HIF-1α axis is the main pathway that affects Th17 responses in Bi2-treated cells.

### HMB regulates HIF-1a via mTORC1 activation in human CD4^+^ T cells

**Fig. 5:**
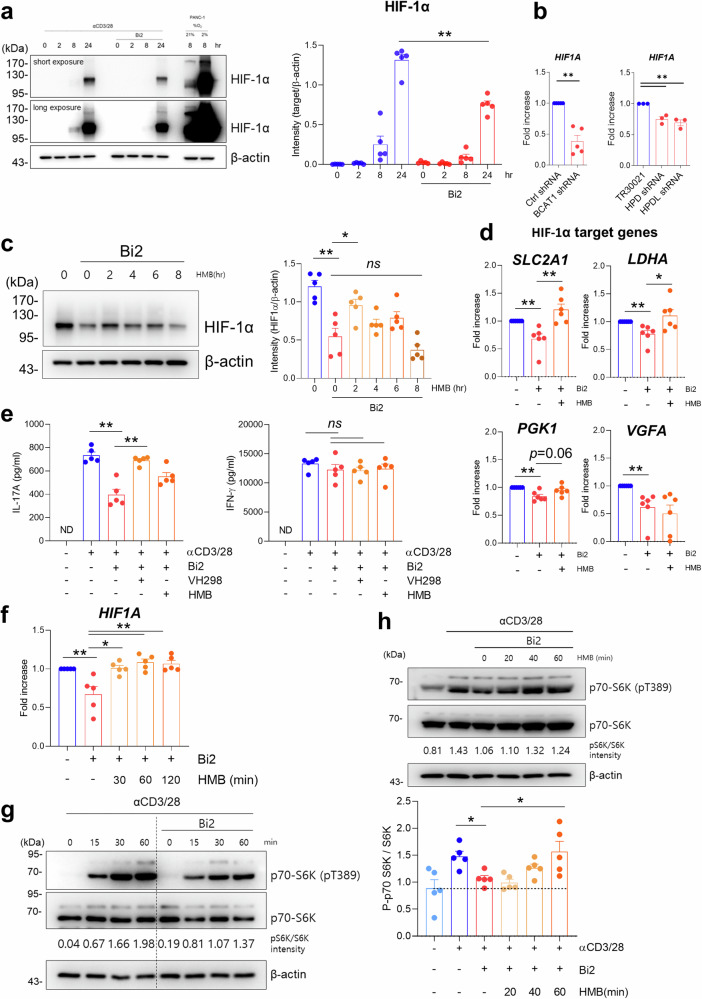
HMB regulates HIF-1α expression in human CD4^+^ T cells. **a** The protein expression of HIF-1α in TCR-stimulated CD4^+^ memory T cells treated with or without Bi2 was analyzed (*n* = 5). As a control for HIF-1α, PANC-1 cells were incubated for 8 h under 2% or 21% O_2_. A representative immunoblot is shown (left). The band intensity in the immunoblots was quantified by densitometry, except for the PANC-1 data. β-Actin was used as a normalization control (right). **b** Freshly isolated human CD4^+^ memory T cells were activated and infected with GFP lentivirus containing BCAT1, HPD, or HPDL shRNA for 24 h. shRNA^+^ cells expressing GFP were sorted and cultured for another 3 days with TCR stimulation. The mRNA expression of HIF-1α was analyzed by RT‒qPCR (*n* = 3–5). **c** HIF-1α expression in human CD4^+^ memory T cells was measured 24 h after TCR stimulation with Bi2 (*n* = 5). The cells were supplemented with HMB (0.4 mM) for the indicated time before harvest. **d** Quantitative PCR analysis of HIF-1α target gene expression after 72 h of TCR stimulation in human CD4^+^ memory T cells from HCs (*n* = 6). HMB (0.4 mM) was used to pretreat the cells for 1 h before TCR stimulation. **e** IL-17A and IFN-γ production were quantified at 72 h after TCR stimulation with or without Bi2 (10 μM), VH298 (100 nM), or HMB (0.4 mM) (*n* = 5). **f** mRNA expression of HIF-1α was measured at 4 h after TCR stimulation. The cells were supplemented with HMB (0.4 mM) for the indicated time before harvest (*n* = 5). **g** CD4^+^ memory T cells were stimulated with anti-CD3/CD28-coated microbeads for 1 h in the absence or presence of Bi2. Cell lysates were prepared at the indicated times and immunoblotted with phospho-p70-S6K and total p70-S6K (*n* = 3 independent experiments). **h** CD4^+^ memory T cells were stimulated with anti-CD3/CD28 Abs for 1 h. The cells were supplemented with HMB (0.4 mM) for the indicated time before harvest. Cell lysates were prepared at 1 h poststimulation and immunoblotted for phospho-p70-S6K and total p70-S6K (*n* = 5 independent experiments). The graph shows the band intensity quantified by densitometry. The graphs show the means ± SEMs. *p < 0.05 and **p < 0.01 according to the Mann‒Whitney *U* test [**a**, **b** (left), **c**–**f**, **h**] or two-tailed unpaired *t* test [**b** (right)].

TCR stimulation induces HIF-1α expression in human CD4^+^ memory T cells at 24 h poststimulation (Fig. 5a). This induction was significantly repressed by blockade of cytosolic leucine metabolism with Bi2 or silencing of BCAT1, HPD, and HPDL with shRNA (Fig. 5a, b). HMB supplementation reversed the Bi2-mediated reduction in HIF-1α (Fig. 5c), and this effect gradually diminished up to 8 h after HMB supplementation. This finding was corroborated by qPCR analysis of the expression of Hif-1α target genes (Fig. 5d). The expression of *SCL2A1, LDHA*, and *PGK1* mRNAs was reduced by Bi2, and this reduction was abrogated by HMB supplementation. We next investigated whether Bi2- or HMB-mediated changes in HIF-1α expression contribute to IL-17A production in CD4^+^ memory T cells. The decrease in IL-17 production induced by Bi2 was completely restored by VH298, a VHL inhibitor that induces the accumulation of HIF-1α^29^. However, treatment with Bi2, HMB, or VH298 did not affect the production of IFN-γ (Fig. 5e). These findings suggest that HMB increases the production of IL-17A by increasing the expression of HIF-1α in human CD4^+^ memory T cells.

HIF-1α is dynamically regulated through a balance of transcription, translation, and degradation^30^. To further understand the mechanisms underlying BCAT1-mediated regulation of HIF-1α, the degradation pathway of HIF-1α was blocked with several inhibitors, such as CoCl_2_, an inhibitor of HIF-1α hydroxylation; VH298, an inhibitor of the E3 ubiquitin ligase pVHL; and MG132, a proteasome inhibitor. (Supplementary Fig. 6a, b). As expected, each inhibitor led to the accumulation of HIF-1α in TCR-stimulated CD4^+^ memory T cells. However, none of these inhibitors affected the reduced expression of HIF-1α caused by Bi2. Thus, we tested whether the HMB-mediated regulation of Hif-1α is attributable to translational control. After depletion of endogenous HMB by pretreatment with Bi2 for 24 h, CD4^+^ memory T cells were treated with cycloheximide (CHX), a protein synthesis inhibitor, in the absence or presence of HMB, and the rate of HIF-1α protein degradation was analyzed (Supplementary Fig. 6c). Consistent with the findings shown in Fig. 5c, HMB supplementation increased the expression of HIF-1α but did not influence the rate of HIF-1α degradation, suggesting that the HMB-mediated increase in HIF-1α is dependent on transcriptional regulation. qPCR revealed that HIF-1α mRNA expression was decreased by Bi2 treatment, and this reduction was rapidly abrogated by HMB supplementation in human CD4^+^ memory T cells (Fig. 5f). In human CD4^+^ T cells, TCR-induced mTORC1 activity results in HIF-1α protein accumulation *via* increased HIF-1α mRNA expression^10,31^. Thus, the activity of p70-S6K, a downstream molecule of mTORC1, was monitored after Bi2 treatment. Our immunoblot assay illustrated that the inhibitory effect of Bi2 treatment on S6K phosphorylation in CD4^+^ memory T cells was observed at approximately 1 to 2 h, relatively early after stimulation (Supplementary Fig. 6d and Fig. 5g). This repression of mTORC1 activity by Bi2 was time-dependently counteracted by HMB supplementation (Fig. 5h). These data demonstrated that HMB, a BCAT1-mediated leucine metabolite, increases mTORC1-dependent HIF-1α expression in CD4^+^ memory T cells.

HIF-1α is also a critical transcription factor in the differentiation program of naive CD4^+^ T cells^32^. Due to the TCR-induced upregulation of SLC7A5 and BCAT1 mRNA expression in human naive CD4^+^ T cells (Fig. 2a), purified naive CD4^+^ T cells were differentiated into Th1 or Th17 cells under in vitro polarizing conditions. Like in CD4^+^ memory T cells, BCAT1 inhibition reduced the activity of mTORC1 and the expression of HIF-1a (Supplementary Fig. 6e, f). In addition, treatment with Bi2 did not significantly affect the viability of activated human CD4^+^ naive T cells (Supplementary Fig. 6g). Our findings indicate an upregulation in the mRNA expression of BCAT1 specifically in Th17-polarized cells, which is indicative of intensified cytosolic leucine metabolic activity (Supplementary Fig. 6h). Consequently, under Th17-polarizing conditions, Bi2 treatment significantly decreased the mRNA levels of *RORC* and *IL17A* and, consequently, reduced IL-17A secretion (Supplementary Fig. 6i, j). Moreover, *IFNG* and *TBX21* mRNA expression and IFN-γ secretion by in vitro polarized Th1 cells were also reduced by BCAT1 inhibition (Supplementary Fig. 6k, l). Collectively, these data revealed that HMB, a cytosolic leucine metabolite, upregulates the expression of HIF-1α through an increase in mTORC1 activity, which is closely associated with the regulation of CD4 T-cell differentiation and functionality.

### Inhibition of BCAT1 ameliorates EAE induction

**Fig. 6:**
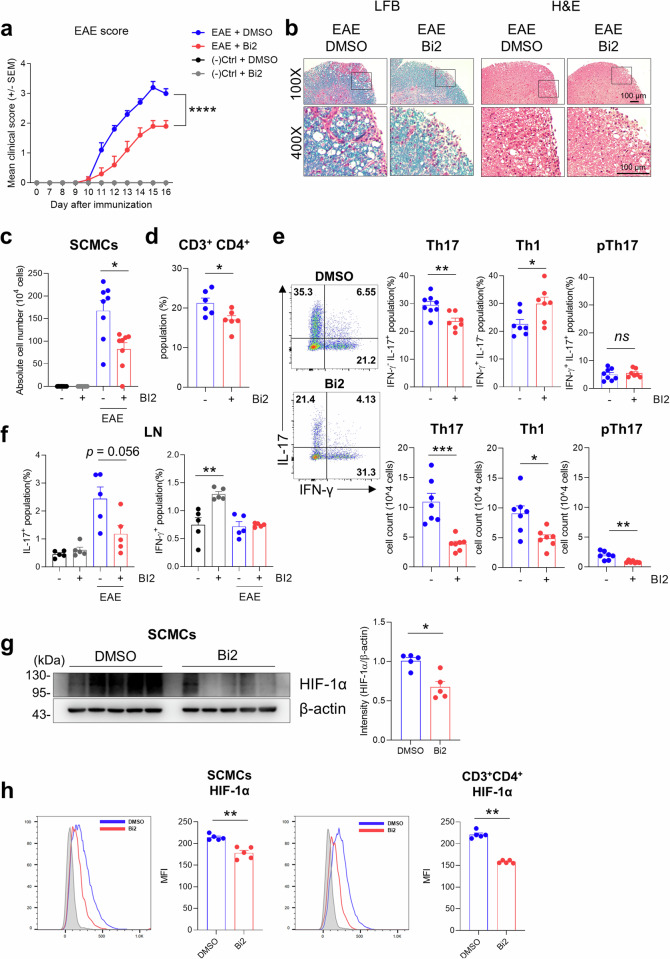
Inhibition of BCAT1 ameliorates EAE severity. Experimental autoimmune encephalomyelitis (EAE) was induced by MOG_35–55_ in a CFA emulsion with PTX. Bi2 (10 mg/kg) was intraperitoneally administered to MOG-immunized mice 4 h before immunization, and the treatment was repeated three times per week for 14 days. **a** Clinical scores of EAE mice. **b** Histological analysis of spinal cord tissue stained with Luxol fast blue or Hematoxylin & Eosin on Day 16. The areas of demyelination (left) and inflammatory cell infiltration (right) are marked with dashed black lines. **c** Quantification of immune cell infiltration in the spinal cord (SCMCs: spinal cord mononuclear cells) among the four different groups. **d** The proportion of CD3^+^CD4 ^+^ T cells among total CD45^+^ T cells in untreated or Bi2-treated EAE mice (*n* = 6 per group). **e**, **f** Flow cytometric analysis of IL-17A- and IFN-γ-producing CD3^+^CD4^+^ T cells in the SCMCs and inguinal lymph nodes (iLNs) of EAE mice (*n* = 5–8). **g** Cell lysates were prepared from SCMCs from DMSO- or Bi2-treated EAE mice and immunoblotted for HIF-α (*n* = 5 per group). **h** A representative histogram of intracellular HIF-1α in total CD45^+^ SCMCs (left) and CD3^+^CD4^+^ T cells (right) from EAE mice (*n* = 5 per group). The graphs show the means ± SEMs. *p < 0.05, **p < 0.01, ***p < 0.001, and ****p < 0.0001 by two-way ANOVA (**a**) or the Mann‒Whitney *U* test (**c**–**h**).

Consistent with the findings observed in human CD4^+^ T cells, TCR-stimulated T cells increased the mRNA expression of *slc7A5* and *bcat1* and the protein expression of BCAT1 in mice (Supplementary Fig. 7a, b). Bi2 treatment preferentially inhibited the differentiation of Th17 cells but not IFN-γ production under each type of Th cell polarizing condition (Supplementary Fig. 7c, d). To examine the systemic in vivo effect of BCAT1 inhibition, we adopted an experimental autoimmune encephalomyelitis (EAE) model widely utilized for studying the pathogenic responses of autoreactive Th17 cells^33^. Bi2 was intraperitoneally administered to MOG-immunized mice 4 h before immunization, and the treatment was repeated three times per week for 14 days (Fig. 6a). Compared with control mice, Bi2-treated mice showed significantly lower clinical scores from Day 12 after EAE induction, and this reduction was maintained until sacrifice (Fig. 6a). Consistent with the changes in clinical score and total weight (Fig. 6a and Supplementary Fig. 8a), histological analysis of the spinal cord tissue revealed that neuronal damage (Fig. 6b) and cellular infiltration (Fig. 6c) were diminished in the Bi2-treated mice. Compared with those in control mice, the absolute number of spinal cord mononuclear cells (SCMCs) and the proportion of CD4^+^ T cells in SCMCs from EAE mice were significantly lower following Bi2 treatment (Fig. 6c, d, and Supplementary Fig. 8b, c). More importantly, Bi2 treatment preferentially reduced the proportion and absolute number of IL-17A-producing CD4^+^ T cells among spinal cord-infiltrating and draining lymph node (LN) CD4^+^ T cells (Fig. 6e, f). Bi2 treatment did not change the relative proportion of pathogenic Th17 cells coproducing IFN-γ/IL-17A; however, their absolute number significantly decreased (Fig. 6e), suggesting that BCAT1 inhibition ameliorates EAE induction by regulating the Th17 responses of CD4^+^ T cells in the draining LN and CNS. In agreement with the findings in human CD4^+^ T cells, TCR stimulation increased HIF-1α expression in mouse LN T cells, and Bi2 treatment diminished the TCR-stimulated increase in HIF-1α expression (Supplementary Fig. 7e, f). Immunoblot analysis revealed that SCMCs isolated from mice administered Bi2 exhibited reduced HIF-1α expression (Fig. 6g). Importantly, intracellular staining for HIF-1α revealed that this reduction preferentially occurred in CD4^+^ T cells but not in CD11b^+^F4/80^+^ macrophages among SCMCs (Fig. 6h and Supplementary Fig. 8d). These data demonstrate that HIF-1α regulation by BCAT1 in CD4^+^ T cells is involved in EAE induction *via* Th17 cell generation.

### LβhL, a leucine analog, attenuates IL-17A production

**Fig. 7:**
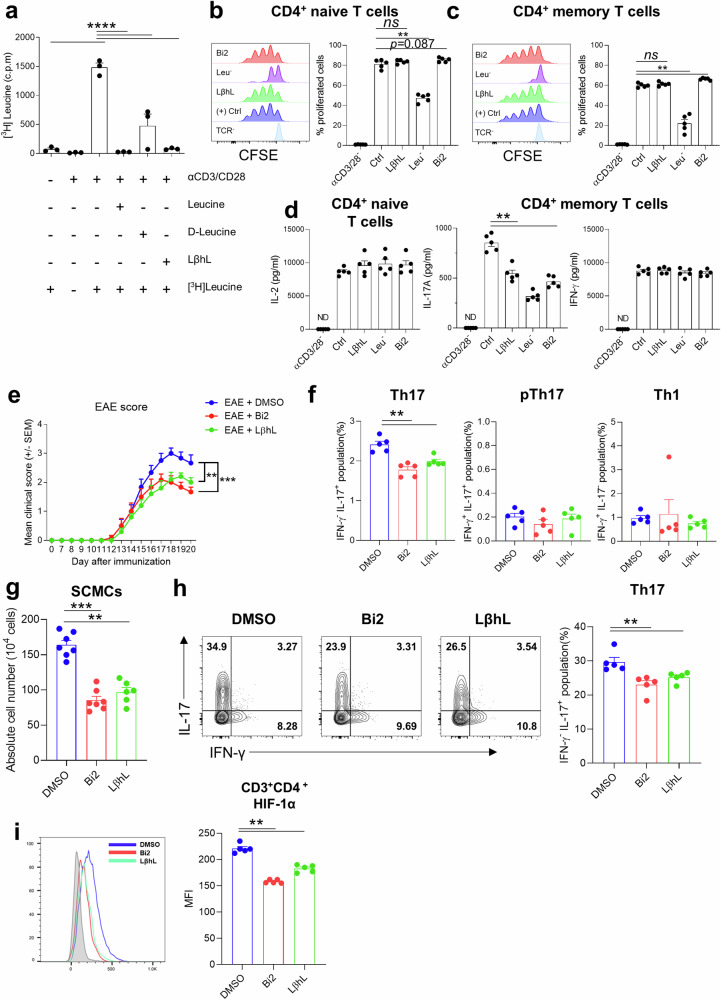
LβhL, a leucine analog, attenuates IL-17A production. **a** Uptake of ^3^H-leucine by TCR-stimulated CD4^+^ T cells in the presence of L-leucine, D-leucine, or LβhL (400 mg/L for each) (*n* = 3). **b**, **c** CFSE-labeled CD4^+^ naive (**b**) and memory (**c**) T cells were stimulated with anti-CD3/CD28-coated microbeads with or without L-leucine, LβhL (400 mg/L), or Bi2 (10 μM) (*n* = 5). The proportion of proliferating cells was measured by CFSE dilution assay. **d** The amount of cytokines in culture supernatant from TCR-stimulated CD4^+^ naive T cells (left) and CD4^+^ memory T cells (right) stimulated with anti-CD3/CD28-coated microbeads for 3 days under the indicated conditions (*n* = 5). Cells were treated with Bi2 and LβhL for 1 h before TCR stimulation. **e** Clinical scores of EAE mice. Bi2 (10 mg/kg) or LβhL (1 g/kg) was intraperitoneally administered to MOG-immunized mice 4 h before immunization, and the treatment was repeated three times per week for 14 days. **f** Flow cytometry analysis of IL-17A- and IFN-γ-producing CD3^+^CD4^+^ T cells in the iLN of EAE mice (*n* = 5 per group). **g** Quantification of SCMCs from EAE mice in the three groups (*n* = 6–7 per group). **h** Flow cytometry analysis of IL-17A- and IFN-γ-producing CD3^+^CD4^+^ T cells in the SCMCs of EAE mice (*n* = 5 per group). **i** A representative histogram plot of intracellular HIF-1α in CD3^+^CD4^+^ T cells among CD45^+^ SCMCs from EAE mice (*n* = 5 per group). The graphs show the means ± SEMs. **p < 0.01, ***p < 0.001, and ****p < 0.0001 by one-way ANOVA with Tukey’s test (**a**), the Mann‒Whitney *U* test (**b**, **c**–**f**, **g**–**i**), or two-way ANOVA (**e**).

Because Bi2 treatment affects BCAA metabolism through BCAT1 inhibition in the EAE mouse model, we next sought to confirm the role of cytosolic leucine bioavailability in regulating Th17 responses in this model. To this end, rather than an SLC7A5 inhibitor, which suppresses the influx of several essential amino acids, including leucine, a leucine analog was utilized as a competitive inhibitor^34^. L-β-homoleucine (LβhL) is a leucine analog that is efficiently transported by SLC7A5 but has a minimal effect on mTORC1 activity^35^. Moreover, LβhL is known to be stable enough for in vivo administration^36^. The incorporation of ^3^H-labeled leucine showed that LβhL competitively inhibited leucine influx in TCR-stimulated CD4^+^ T cells (Fig. 7a). Similar to the inhibition of BCAT1 by Bi2 treatment, LβhL had no effect on the proliferation of human CD4^+^ T cells (Fig. 7b, c), whereas LβhL preferentially decreased the production of IL-17A, but not IFN-γ, by CD4^+^ memory T cells and IL-2 by CD4^+^ naive T cells in humans (Fig. 7d). As shown in Fig. 7e, the induction and severity of EAE were attenuated in LβhL-treated mice compared with control mice to a level comparable to that in Bi2-treated mice (Fig. 7e). This finding was also confirmed by the histological analysis of spinal cord tissue, which revealed neuronal damage (Supplementary Fig. 9), and cellular infiltration (Fig. 7g) was significantly lower in the LβhL-treated group than in the control group. Flow cytometry analysis demonstrated that LβhL treatment predominantly reduced the proportion of IL-17A-producing CD4^+^ T cells among spinal cord-infiltrating (Fig. 7h) and draining LN CD4^+^ T cells (Fig. 7f). More importantly, HIF-1α expression was decreased in CD4^+^ T cells from the SC of LβhL-treated mice (Fig. 7i), similar to what was observed in Bi2-treated mice. These data highlight that activation-induced leucine influx and BCAT1-mediated leucine metabolism in CD4^+^ T cells are important for Th17 responses that regulate EAE induction.

## Discussion

Accumulating evidence indicates that BCAAs, supplied by SLC7A5, play a crucial role in metabolic rewiring in activated immune cells^37^. In murine Treg cells, isoleucine uptake *via* SLC3A2, which forms a heterodimer with SLC7A5, activates the mTORC1 pathway, leading to altered metabolic states^38^. BCAA accumulation in CD8^+^ T cells enhances glucose uptake through increased Glut1 expression, promoting metabolic reprogramming and antitumor immunity^39^. In macrophages, SLC7A5-mediated leucine influx induces IL-1β production *via* mTORC1-induced glycolytic reprogramming upon LPS stimulation^15^. BCAT1-regulated BCAA catabolism in activated macrophages is associated with inflammatory diseases and itaconate production in the TCA cycle^16^.

Despite being essential for protein synthesis^40^, elevated BCAAs are linked to systemic diseases, including cancer, heart failure, diabetes, and insulin resistance, possibly due to their involvement in the TCA pathway and signaling roles in metabolic pathways^41–43^. Leucine, for instance, activates mTORC1 through direct association with leucine sensors such as leucyl-tRNA synthetase or Sestrin2^44^. Although less well known, leucine-derived metabolites also support metabolic rewiring in activated immune cells^45^. Reprogramming of BCAA metabolism relies on altered expression and activity of metabolic enzymes (e.g., BCATs) and transporters (e.g., SLC7A5). Our findings demonstrated that TCR stimulation increases BCAT and SLC7A5 expression in human CD4^+^ T cells, emphasizing their importance in BCAA metabolism reprogramming.

One interesting observation in our study was that the uptake and catabolism of BCAAs, especially leucine, preferentially influenced IL-17 production in human CD4^+^ memory T cells (Fig. 2c–e and Supplementary Fig. 3c). This prompted us to investigate whether cytoplasmic leucine metabolites catabolized by BCAT1 are involved in the regulation of Th17 responses in CD4^+^ T cells. BCAT transaminates with leucine to form α-KIC with the concomitant production of glutamate from α-ketoglutarate (α-KG). In the mitochondria, α-KIC is mainly oxidized to isovaleryl-CoA by the BCKD complex, leading to the formation of HMG-CoA and its final metabolites, including acetoacetate and acetyl-CoA^46^. An alternative pathway of leucine catabolism has been reported in the cytosol in which α-KIC is converted to β-hydroxy β-methyl butyric acid (HMB) by KIC dioxygenase [also known as 4-hydroxyphenylpyruvate dioxygenase (HPD)]^45^ or 4-hydroxyphenylpyruvate dioxygenase-like protein (HPDL), which has dioxygenase activity similar to that of HPD^46^. Given the demonstrated influence of HMB, a cytosolic leucine metabolite, on the regulation of IL-17A production, the silencing of HPD with shRNA had a comparable impact on the inhibition of BCAT1. Moreover, the verified decrease in IL-17A production upon HPDL knockdown underscores the participation of HPDL, which possesses enzymatic activity analogous to that of HPD, in the modulation of cytosolic leucine metabolism.

HMB is a key metabolite of cytosolic leucine metabolism, and its plasma level is dependent on the consumption of leucine-rich diets^47^. Since HMB can promote muscle anabolism by improving muscle protein synthesis and attenuating muscle protein degradation through mTORC1 activation more effectively than leucine^43,48^, HMB has been used as a dietary substitute for leucine^49^. Notably, HMB-induced mTORC1 activation is independent of the leucine-sensing pathway mediated by Sestrin2^50^. In our study, the production of Th17 cells in CD4^+^ memory T cells was significantly reduced by BCAT1 inhibition with Bi2, and this reduction was abrogated by exogenous HMB, suggesting that HMB plays a regulatory role in Th17 responses (Fig. 3g). In the cytosolic leucine metabolic pathway, HMB can be further metabolized into HMG-CoA, which participates in de novo cholesterol synthesis^26^. Considering that cholesterol intake and synthesis are important for Th17 differentiation and responses^51^, the reduced IL-17 production observed in response to Bi2 could be due to a decrease in cholesterol synthesis. As expected, statin, a pharmacological inhibitor of HMG-CoA reductase, the rate-limiting enzyme for de novo cholesterol synthesis, reduced intracellular cholesterol levels and decreased IL-17A production (Fig. 3d, e). However, Bi2 treatment markedly increased intracellular cholesterol levels in TCR-activated CD4^+^ memory T cells, while exogenous HMB decreased cholesterol levels in these cells (Fig. 3d). These data suggest that BCAT1-mediated leucine metabolism does not influence the synthesis of intracellular cholesterol and that the regulation of IL-17A by cytosolic leucine metabolism is independent of de novo cholesterol synthesis in CD4^+^ memory T cells in humans.

Cytosolic leucine metabolism also supports the synthesis of de novo acetyl-CoA, which is reversibly converted from HMG-CoA^46^. Although acetyl-CoA is known to support the supply of fatty acids to proliferating T cells^52^ and enhance Th17 responses *via* epigenetic reprogramming^28,53^, our data demonstrated that BCAT1 inhibition had no effect on CD4^+^ T-cell proliferation (Fig. 2b) and that supplementation with acetyl-CoA did not restore IL-17 production following BCAT1 inhibition (Fig. 3f). These data support the idea that HMB directly contributes to the regulation of IL-17 production. The concentration (400 μM) of HMB used in the present study is an achievable plasma level following oral administration of HMB in healthy adult humans. These HMB plasma levels peak within 60 to 120 min, and the plasma half-life is ~2.5 h^54^. Considering that exogenous HMB treatment for 1 h counteracts the repression of mTORC1 activity by Bi2 (Fig. 5g), it is also possible that HMB supplementation affects T-cell responses in humans. A study by Ananieva and colleagues revealed the hyperactivation of T cells in BCAT1^-/-^ mice, which were characterized by increased glycolytic metabolism and increased ATP synthesis capacity. Furthermore, these T cells exhibit diminished leucine transamination and elevated intracellular leucine concentrations, potentially enhancing the activity of the mTORC1/4EBP1 signaling pathway. Consequently, BCAT1 is posited to be a component of a negative feedback loop governing leucine availability for mTORC1 regulation in T cells^12^. In contrast, our investigation revealed that inhibition of BCAT1 with a chemical agent does not induce hyperactivation of human CD4 + T cells upon TCR stimulation (Fig. 2b–f). Although the precise mechanism underlying this disparity remains elusive, our study demonstrated the restoration of leucine-dependent Th17 production in CD4^+^ memory T cells through leucine supplementation at concentrations as low as 10 mg/L, mirroring the physiological levels of leucine in human plasma (Fig. 2c). It is plausible that the concentration of intracellular leucine required for direct mTORC1 activation may be constrained, and the involvement of mTORC1 activity in Th17 production in human T cells may involve both intracellular leucine and its metabolites *via* an HIF1α-dependent pathway. Recent investigations have shown the inhibitory effects of pharmacological BCAT1 inhibition using novel drugs on the effector functions of human CD8^+^ T cells in vitro. Notably, such inhibition leads to a metabolic shift toward enhanced OXPHOS, underscoring the importance of BCAT1 in the differentiation of CD8^+^ T cells and their effector functions, including cytokine production^55^.

Our scRNA-seq analysis supported the findings that the inhibition of cytosolic leucine metabolism with Bi2 has a predominant impact on activated Th17 clusters and the mTORC1-HIF1α axis (Fig. 4). HIF1α is a crucial signaling molecule in immune responses, and its activity is coordinately regulated by a balance of transcription, translation, and degradation. Immune cell activation, such as TCR or TLR engagement, increases HIF1α expression *via* mTORC1 signaling to induce metabolic rewiring, which is critical for the effector function of immune cells^56^. It has been reported that HIF1α enhances direct transcriptional activation of RORγt and promotes recruitment of the RORγt and p300 complex to the IL-17A promoter, thereby regulating Th17 signature genes^32^. In the EAE mouse model, HIF1α^-/-^ CD4 T cells are deficient in IL-17A production but not in IFN-γ production in the spleen or lymph nodes, indicating resistance to EAE^32^. Here, we found that BCAT1 inhibition attenuates TCR-induced HIF1α expression in CD4^+^ memory T cells and that exogenous HMB abrogates this attenuation at the transcriptional level through enhancing the activity of the mTORC1/S6K axis (Fig. 5a, b, e), leading to increased expression of HIF1α target genes and the production of IL-17A (Fig. 5c, d). A critical role of HIF1α in the reduced production of IL-17 was suggested by the finding that VH298, an inhibitor of the VHL-HIF-α interaction, abrogates the decrease in IL-17A following BCAT1 inhibition (Fig. 5d).

In conclusion, the current study provides new insight into the role of cytosolic leucine metabolism in modulating CD4^+^ T-cell responses, especially IL-17 production, by investigating the effect of inhibitors of leucine-specific transporters or catabolizing enzymes in vivo and in vitro. Upon TCR stimulation, the cytosolic leucine metabolic pathway is activated by SLC7A5-mediated influx and BCAT1-mediated metabolism in CD4^+^ memory T cells, contributing to enhanced production of IL-17A. Our data suggest that HMB, a cytosolic leucine metabolite, is involved in augmenting IL-17 production *via* the upregulation of mTORC1-dependent HIF1α expression. Consistent with our in vitro findings, the EAE mouse model showed that blockade of BCAT1-mediated leucine catabolism by treatment with a BCAT1 inhibitor or a leucine analog ameliorates EAE severity by decreasing HIF1α expression and IL-17 production in CD4^+^ T cells in the spinal cord. These results suggest that the modulation of cytosolic leucine metabolism through BCAT1 inhibition is a potential therapeutic approach for the treatment of Th17-related inflammatory diseases.

## Supplementary information

Supplementary table 3

Supplementary figures 1-9 and tables 1-2
