# Supplementary material for: Inhibition of BCAT1-mediated cytosolic leucine metabolism regulates Th17 responses via the mTORC1-HIF1α pathway
Source: Exp Mol Med. 2024 Aug 1;56(8):1776–90. doi: 10.1038/s12276-024-01286-z (PMC11372109; doi:10.1038/s12276-024-01286-z)
Supplement: Supplementary file 2 — Supplementary figures 1-9 and tables 1-2 [file 12276_2024_1286_MOESM2_ESM.pdf]

Inhibition of BCAT1-mediated cytosolic leucine metabolism regulates Th17 responses *via* the mTORC1-HIF1α pathway

Yeon Jun Kang, Woorim Song, Su Jeong Lee, Seung Ah Choi, Sihyun Chae, Bo Ruem Yoon, Hee Young Kim, Jung Ho Lee, Chulwoo Kim, Joo-Youn Cho, Hyun Je Kim, and Won-Woo Lee

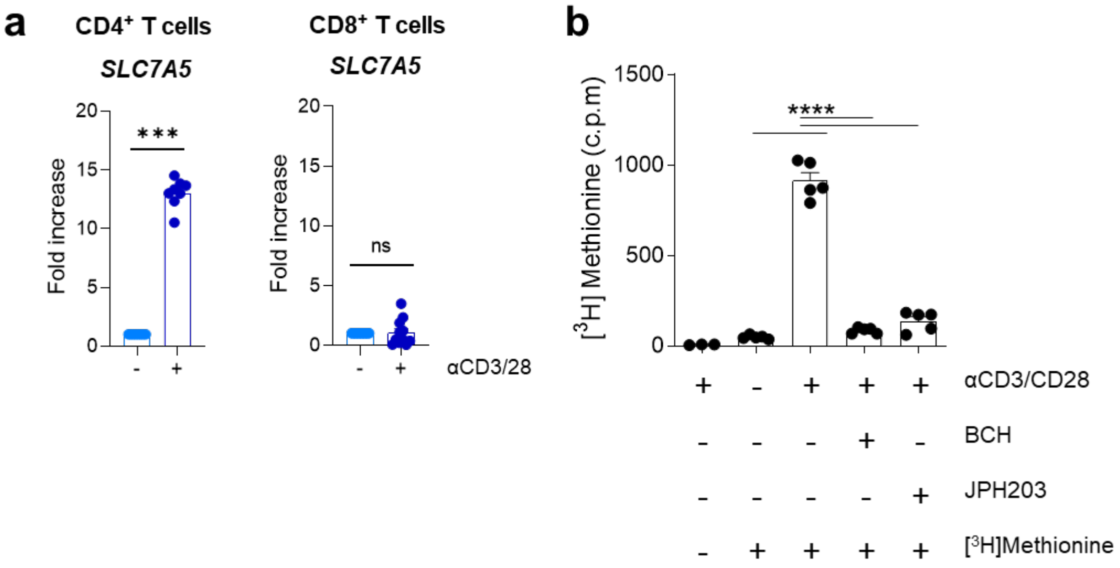

Supplementary Fig. 1. mRNA expression of SLC7A5 is preferentially induced by TCR-stimulated CD4 T cells. a. mRNA expression of SLC7A5 was analyzed by RT-qPCR at 24 h after TCR stimulation in human $CD4^+$ T cells and $CD8^+$ T cells from HCs (n = 8~11). **b.** Uptake of ^{3}H-methionine by TCR-stimulated human $CD4^+$ T cells in the presence of BCH or JPH203 (n = 5). Graphs show mean ± SEM. *** = $p < 0.001$ and **** = $p < 0.0001$ by Mann-Whitney U test (a) or one-way ANOVA with Turkey's (b).

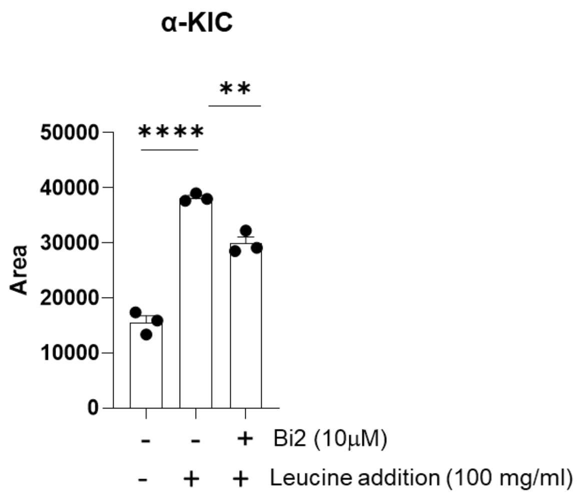

Supplementary Fig. 2. The impact of BCAT1 inhibition (Bi2) on pharmacological responses in HepG2 cells. Cellular levels of α-ketoisocaproic acid (α-KIC), a metabolite of BCAT, were assessed in HepG2 cells under conditions of leucine supplementation or treatment with Bi2. Quantitative representation of α-KIC levels are depicted (n = 3). Graphs show the mean ± SEM. **= $p < 0.01$ and **** = $p < 0.0001$ by two-tailed unpaired *t*-test.

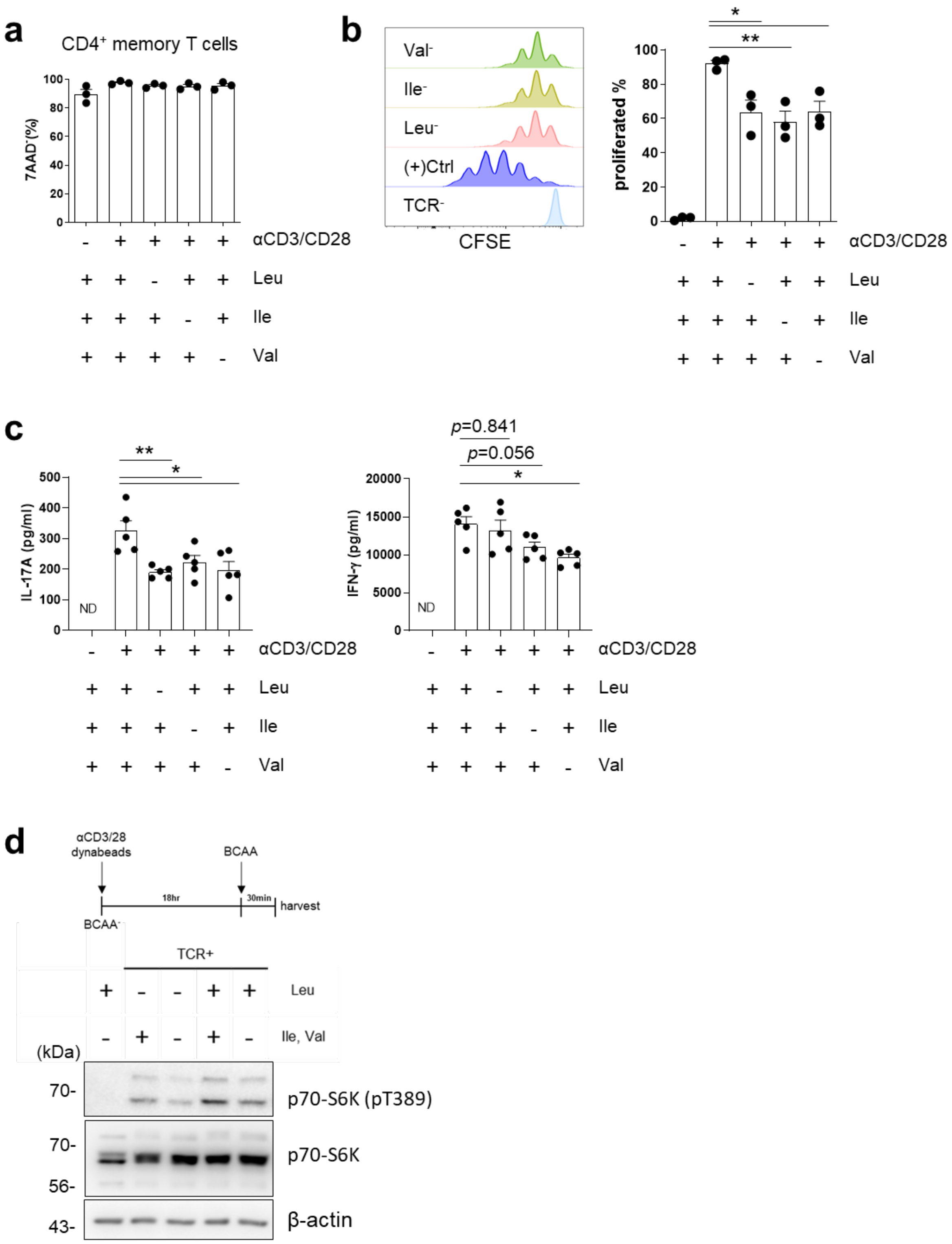

Supplementary Fig. 3. BCAAs are involved in the regulation of effector functions of CD4⁺ memory T cells. a-c. CFSE labeled or non-labeled CD4⁺ memory T cells were stimulated with anti-CD3/CD28-coated microbeads in leucine-, isoleucine-, or valine-depleted media for 5 days (n = 3 ~ 5). Cell viability (a) was analyzed by 7-AAD staining using flow cytometry (n = 3). The proportion of proliferating cells (b) was measured by

CFSE dilution assay (n = 3). The amount of IL-17A and IFN-γ (c) in culture supernatant from TCR-stimulated CD4⁺ memory T cells under the indicated conditions was measured by ELISA (n = 5). **d**. CD4⁺ memory T cells were stimulated with anti-CD3/CD28-coated microbeads for 18 hr in BCAA-depleted media. Cells were supplemented with leucine, isoleucine, or valine for 30 min before harvest. Cell lysates were prepared and immunoblotted with phosphor-p70-S6K and total p70-S6K (n=3 independent experiments). Graphs show the mean ± SEM. *= $p < 0.05$ and **= $p < 0.01$ by two-tailed unpaired t-test (b) or Mann-Whitney U test (c).

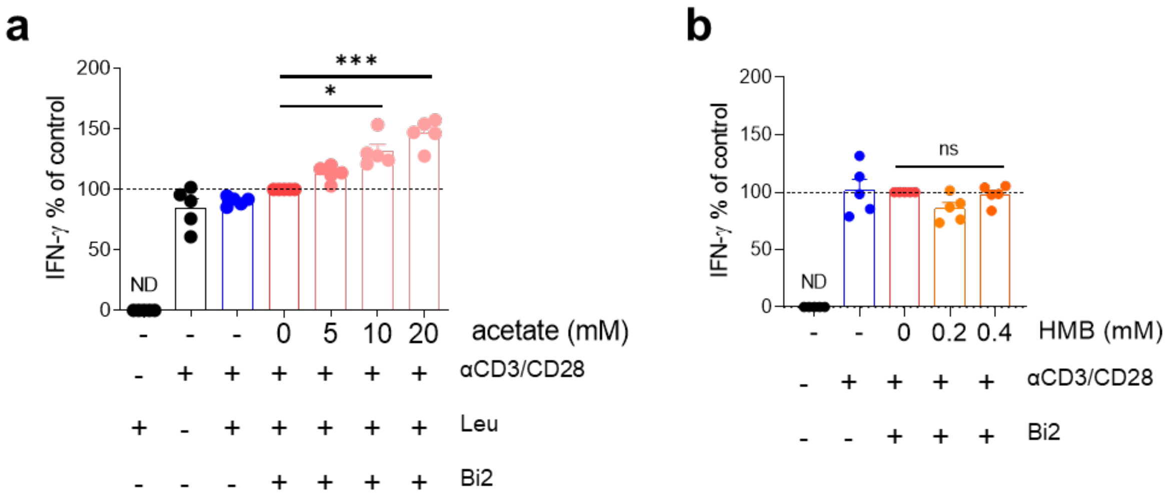

Supplementary Fig. 4. BCAT1-mediated leucine metabolites have a different effect on IFN-γ production of CD4⁺ memory T cells compared to IL-17A production. a. TCR-stimulated CD4⁺ memory T cells were cultured for 3 days with Bi2 (10 μM) and the indicated concentration of acetate. The amount of IFN-γ was measured by ELISA (n = 5). **b.** The amount of IFN-γ in culture supernatant from CD4⁺ memory T cells with HMB supplementation was measured by ELISA (n = 5). Graphs show the mean ± SEM. *= $p < 0.05$ and *** = $p < 0.001$ by one-way ANOVA with Kruskal Wallis test.

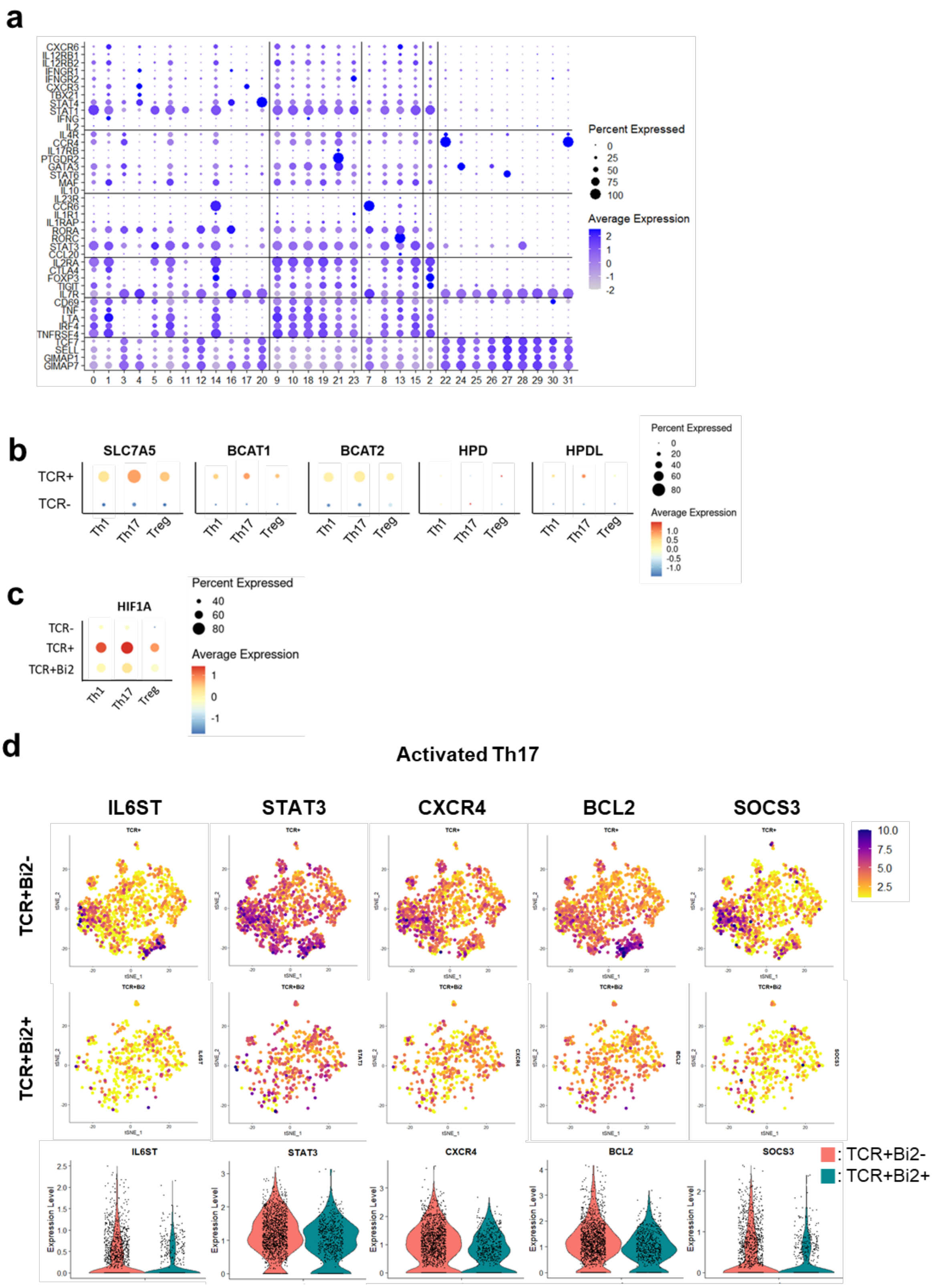

Supplementary Fig. 5. Blockade of BCAT1 is associated with distinct transcriptomic profiles in activated Th17 T cells. a. Dot-plot shows scaled average gene expression of the

major marker genes of CD4$^+$ memory T cell in each cluster of (Fig. 4A). Colors indicate expression, while the size of the circles represents the proportion of expressed cells. **b and c.** The expression patterns of leucine metabolism-related genes across different subsets of CD4$^+$ T cells. Dot plots depict the scaled average gene expression of each gene (b) and HIF1α (c) within CD4$^+$ memory T cell populations categorized into Th1, Th17, or Treg clusters (refer to Figure 4a in the revised manuscript). Colors represent expression levels, while circle size indicates the proportion of cells expressing the respective gene. **d.** Expression of the major marker genes of activated Th17 cells was projected onto the *t*-SNE plot shown as FeaturePlots (top). Violin plots show the distribution of expression levels of the genes and dots represent individual cells (bottom).

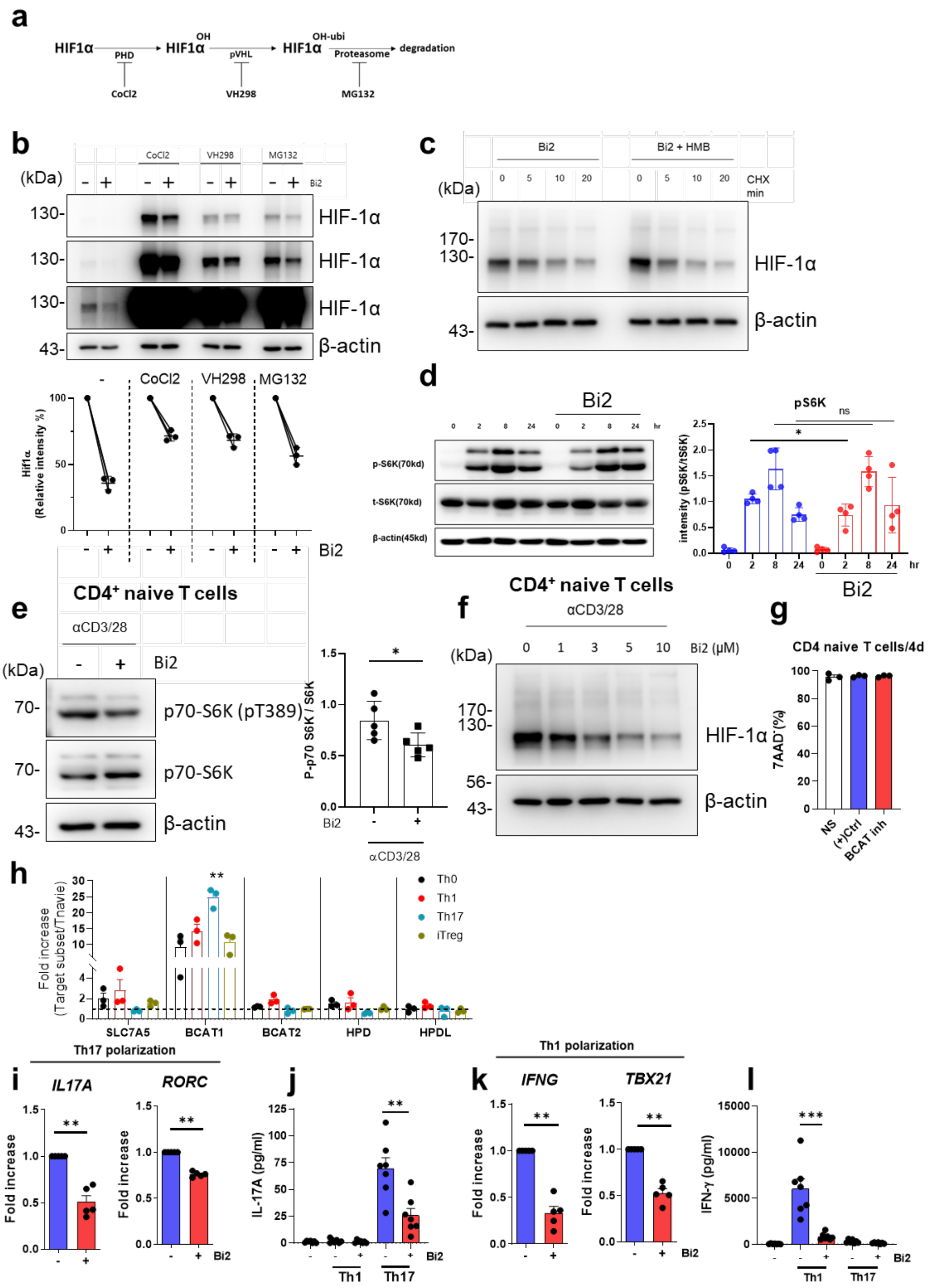
a
HIF1α
PHD
CoCl2
OH
HIF1α
pVHL
VH298
OH-ubi
HIF1α
Proteasome
MG132
degradation
b
(kDa)
CoCl2
VH298
MG132
Bi2
130-
HIF-1α
43-
β-actin
Hif1α (Relative intensity %)
c
Bi2
Bi2 + HMB
CHX min
170-
d
p-S6K(70kd)
t-S6K(70kd)
β-actin(45kd)
pS6K
ns
intensity (pS6K/tS6K)
hr
e
CD4+ naive T cells
αCD3/28
70-
p70-S6K (pT389)
p70-S6K
P-p70 S6K / S6K
f
Bi2 (μM)
56-
g
CD4 naive T cells/4d
7AAD- (%)
NS
(+)Ctrl
BCAT inh
h
Fold increase (Target subset/Tnavie)
Th0
Th1
Th17
iTreg
SLC7A5
BCAT1
BCAT2
HPD
HPDL
Th17 polarization
i
IL17A
RORC
Fold increase
j
IL-17A (pg/ml)
Th1
Th17
Th1 polarization
k
IFNG
TBX21
l
IFN-γ (pg/ml)

Supplementary Fig. 6. BCAT1 regulates HIF-1α *via* mTORC1 activation in human CD4⁺ T cells. a. Ubiquitin-proteasome degradation of HIF1a and an inhibitor for each step. **b.** $CD4^+$ memory T cells were pre-treated with Bi2 (10 μM), $CoCl_2$ (100 μM), VH298 (100 μM), or MG132 (5 μM) for 1 hr and stimulated with anti-CD3/CD28-coated microbeads for 8 hrs. Cell lysates were immunoblotted for HIF-1α (n=3 independent experiments). **c**, $CD4^+$ memory T cells were stimulated for 24 hr, followed by treatment with CHX (0.2 μg/ml) for the indicated time before harvest. Cell lysates were immunoblotted for HIF-1α (n=3 independent experiments). **d.** $CD4^+$ memory T cells were stimulated for the indicated times with or without Bi2 (10 μM). Cell lysates were immunoblotted for phosphor-p70-S6K and total p70-S6K (n = 4 independent experiments). The graph shows the band intensity quantified by densitometry. **e.** $CD4^+$ naive T cells were pretreated with Bi2 (10 μM) for 1 hr and stimulated with anti-CD3/CD28 Abs for 1 hr. Cell lysates were immunoblotted for phosphor-p70-S6K and total p70-S6K (n = 5 independent experiments). **f.** $CD4^+$ naive T cells were stimulated for 24 hr in the presence of the indicated concentration of Bi2. Cell lysates were immunoblotted for HIF-1α (n = 3 independent experiments). **g.** $CD4^+$ naive T cells were stimulated with anti-CD3/CD28-coated microbeads for 4 days with Bi2 (10 μM). Cell viability was analyzed by 7-AAD staining using flow cytometry (n = 3). **h.** Human $CD4^+$ naive T cells were isolated and treated with Bi2 (10 μM) for 1 hour prior to TCR stimulation for differentiation under various cytokine conditions (Th0, Th1, Th17, or iTreg). mRNA levels of genes involved in leucine metabolism were quantified using RT-qPCR (n = 3). **i-l.** $CD4^+$ naive T cells were pretreated with Bi2 (10 μM) for 1 hr and were differentiated under Th17 or Th1 polarizing conditions for 7 days. mRNA expression of signature genes and cytokines of Th17 (**i-j**) or Th1 (**k-l**) were measured by RT-qPCR and ELISA, respectively (n = 5-7). Graphs show mean ± SEM. *= $p < 0.05$, **= $p < 0.01$, and *** = $p < 0.001$ by Mann-Whitney U test.

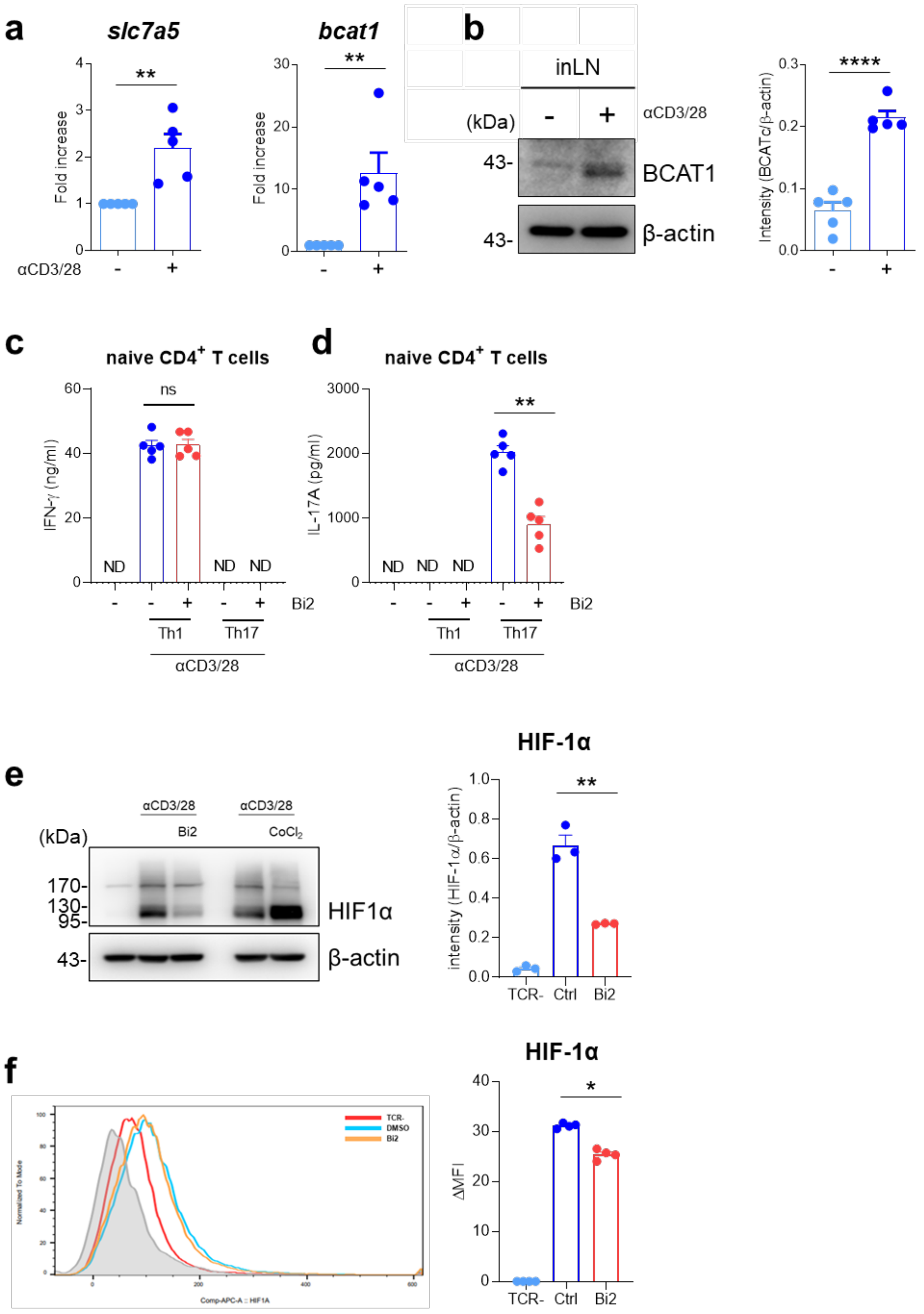

Supplementary Fig. 7. Blockade of BCAT1 with Bi2 attenuates Th17 differentiation in mice. a. mRNA expression of *slc7a5* and *bcat1* in cells from mouse inguinal LN (iLN) was analyzed by RT-qPCR at 24 h after TCR stimulation with anti-CD3/CD28 Abs (n = 5). **b**, Protein expression of BCAT1 in the cells from mouse inguinal LN was analyzed at 24 hr after TCR stimulation with anti-CD3/CD28 Abs (*n* = 5). **c-d.** Mouse $CD4^+$ naive T cells derived from iLN were differentiated under Th17 or Th1 polarizing conditions for 5 days in the presence of Bi2 (10 μM). The amount of IFN-γ (c) and IL-17A (d) in culture supernatant from Th1 and Th17 cells (*n* = 5). **e.** Cells from mouse iLN were stimulated with anti-CD3/CD28 Abs for 24 hr in the presence of Bi2 (10 μM). Cell lysates were immunoblotted for HIF-1α (n = 3 independent experiments). $CoCl_2$-treated cells were used as a positive control of HIF-1α. The graph shows band intensity quantified by densitometry. **f.** A

representative histogram plot of intracellular HIF-1α in the cells from mouse iLN. Cells were stimulated with anti-CD3/CD28 Abs for 24 hr in the presence of Bi2 (10 μM). Graphs show mean ± SEM. *= $p < 0.05$, **= $p < 0.01$, and *** = $p < 0.001$ by Mann-Whitney *U* test (a, c, d, f) or two-tailed unpaired *t*-test (b, e).

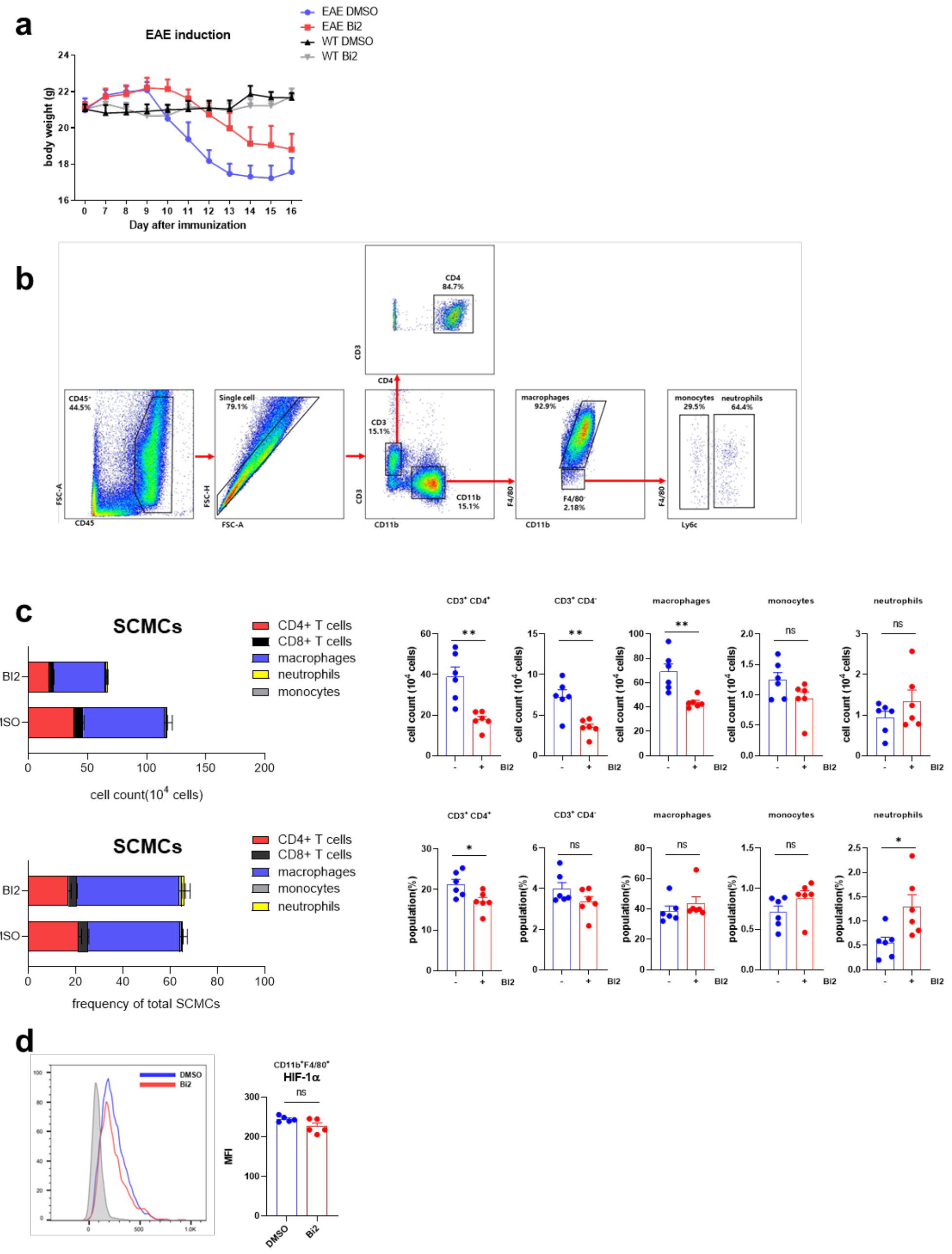

Supplementary Fig. 8. Inhibition of BCAT1 ameliorates EAE induction. Experimental autoimmune encephalomyelitis (EAE) was induced by $MOG_{35–55}$ in CFA emulsion with PTX. Bi2 (10 mg/kg) was intraperitoneally administered to MOG-immunized mice at 4 h before immunization and treatment repeated three times per week until 14 days. **a.** Body weight of mice (*n* = 5 per group). **b.** Gating strategy of flow cytometric analysis to identify different immune cell subsets in the spinal cord mononuclear cells (SCMCs). **c.** The absolute number and the frequency of each subset in SCMCs were analyzed by flow cytometry (*n* = 6 per

group). **d.** A representative histogram plot of intracellular HIF-1α in the $CD11b^{+}F4/80^{+}$ macrophages of $CD45^{+}$ population in SCMCs from EAE mice ($n = 5$ per group). Graphs show mean ± SEM. *= $p < 0.05$ and **= $p < 0.01$ by Mann-Whitney *U* test.

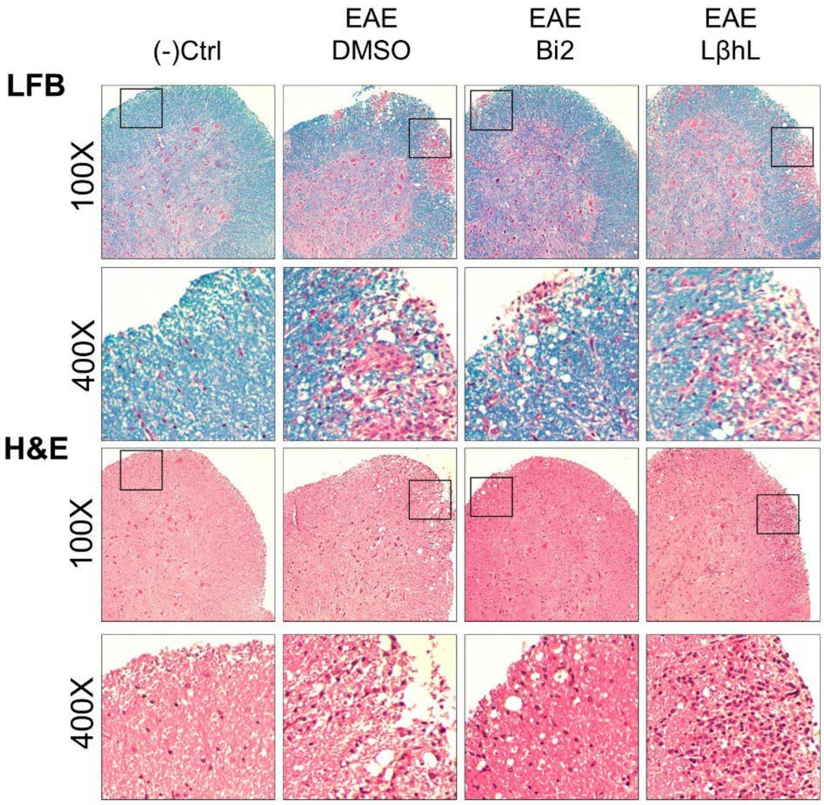

Supplementary Fig. 9. LβhL, a leucine analogue, ameliorates EAE induction to a similar extent to Bi2-treated mice. Histological analysis of spinal cord tissue stained with Luxol fast blue or Hematoxylin & Eosin. The area of demyelination (top) and inflammatory cell infiltration (bottom) were marked with a dashed black line.

Supplementary Table 1. Primers for qPCR.

Gene name	Primer sequence (5'-3')
**human BCAT1**	Forward: AAGATGGGAGGGAATTACGG<br>Reverse: TGGAGGAGTTGCCAGTTCTT
**human BCAT2**	Forward: TTCCAGAAGGAGCTGAAGGA<br>Reverse: CGCTGGCCTTTTATTTCGTA
**human SLC7A5**	Forward: GAAGGCACCAAACTGGATGT<br>Reverse: GAAGTAGGCCAGGTTGGTCA
**human SLC3A2**	Forward: CCAGAAGGATGATGTCGCTCAG<br>Reverse: GAGTAAGGTCCAGAATGACACGG
**human SLC7A8**	Forward: GACAGATAGTCCTTCGCTGGAAG<br>Reverse: TCTGACCACAGGCTGAAGACCA
**human SLC25A44**	Forward: GAGGCTATGTGGCTTCACTGCT<br>Reverse: GACAATGTGAGGGCACTCCTTAG
**human HPD**	Forward: CCCTGGAACAAAGAGATGGGCGAT<br>Reverse: GATTTTGGCGCCCCGTTCCC
**human HPDL**	Forward: CGAAGTCCCCAACCACAAGT<br>Reverse: CGCCCTCGTTCACCAAAAAG
**human BCKDK**	Forward: TCCCCTTCATCCCTATGCCA<br>Reverse: CAGAGACCCACCGAGGTACT
**human HIF1A**	Forward: CCATTAGAAAGCAGTTCCGC<br>Reverse: TGGGTAGGAGATGGAGATGC
**human SLC2A1**	Forward: TTGCAGGCTTCTCCAACTGGAC<br>Reverse: CAGAACCAGGAGCACAGTGAAG
**human LDHA**	Forward: GGATCTCCAACATGGCAGCCTT<br>Reverse: AGACGGCTTTCTCCCTCTTGCT
**human PGK1**	Forward: CCGCTTTCATGTGGAGGAAGAAG<br>Reverse: CTCTGTGAGCAGTGCCAAAAGC
**human VGFA**	Forward: TTGCCTTGCTGCTCTACCTCCA<br>Reverse: GATGGCAGTAGCTGCGCTGATA
**human IL-17A**	Forward: ACTACAACCGATCCACCTCAC<br>Reverse: ACTTTGCCTCCCAGATCACAG
**human RORC**	Forward: TTTTCCGAGGATGAGATTGC<br>Reverse: CTTTCCACATGCTGGCTACA
**human IFNG**	Forward: TGACCAGAGCATCCAAAAGA<br>Reverse: CTCTTCGACCTCGAAACAGC
**human TBX21**	Forward: ATTGCCGTGACTGCCTACCAGA<br>Reverse: GGAATTGACAGTTGGGTCCAGG

**human BACTIN**	Forward: GGACTTCGAGCAAGAGATGG<br>Reverse: AGCACTGTGTTGGCGTACAG
**mouse SLC7A5**	Forward: GGTCTCTGTTCACGTCCTCAAG<br>Reverse: GAACACCAGTGATGGCACAGGT
**mouse BCAT1**	Forward: CTGCCTCTGTTTTGCACTACGC<br>Reverse: TCCTCACAGCAGATCGGCACAT
**mouse BACTIN**	Forward: AGCCATGTACGTAGCCATCC<br>Reverse: CTCTCAGCTGTGGTGGTGAA

Supplementary Table 2. Antibodies for flow cytometric analysis.

Description	Source	Identifiers
Anti-mouse CD45 FITC (Rat monoclonal)	BD Bioscience	553080
Anti-mouse CD3e APC-Cy7 (Hamster monoclonal)	BD Bioscience	557596
Anti-mouse CD4 PE (Rat monoclonal)	BD Bioscience	561837
Anti-mouse CD11b V450 (Rat monoclonal)	BD Bioscience	560455
Anti-mouse CD4 PerCP (Rat monoclonal)	BD Bioscience	553052
Anti-mouse F4/80 PE-Cy7 (Rat monoclonal)	Invitrogen	25-4801-82
Anti-mouse IL-17A Brilliant Violet 421(Rat monoclonal)	BioLegend	506925
Anti-mouse IFN-γ APC (Rat monoclonal)	Invitrogen	17-7311-82
Anti-human/mouse HIF-1α APC (Mouse monoclonal)	R&D Systems	IC1935A
